# VivaxGEN: An open access platform for comparative analysis of short tandem repeat genotyping data in *Plasmodium vivax* populations

**DOI:** 10.1371/journal.pntd.0005465

**Published:** 2017-03-31

**Authors:** Hidayat Trimarsanto, Ernest D. Benavente, Rintis Noviyanti, Retno Ayu Setya Utami, Leily Trianty, Zuleima Pava, Sisay Getachew, Jung-Yeon Kim, Youn-Kyoung Goo, Sonam Wangchuck, Yaobao Liu, Qi Gao, Simone Dowd, Qin Cheng, Taane G. Clark, Ric N. Price, Sarah Auburn

**Affiliations:** 1 Eijkman Institute for Molecular Biology, Jakarta Pusat, Indonesia; 2 Agency for Assessment and Application of Technology, Jakarta Pusat, Indonesia; 3 Faculty of Infectious and Tropical Diseases, London School of Hygiene and Tropical Medicine, London, United Kingdom; 4 The Ministry of Research, Technology and Higher Education, Jakarta Pusat, Indonesia; 5 Global and Tropical Health Division, Menzies School of Health Research and Charles Darwin University, Darwin, Northern Territory, Australia; 6 College of Natural Sciences, Addis Ababa University, Addis Ababa, Ethiopia; 7 Armauer Hansen Research Institute, Addis Ababa, Ethiopia; 8 Division of Malaria and Parasitic Diseases, National Institute of Health, Korea CDC, Osong, Republic of Korea; 9 Department of Parasitology and Tropical Medicine, Kyungpook National University School of Medicine, Daegu, Republic of Korea; 10 Royal Center for Disease Control, Department of Public Health, Ministry of Health, Thimphu, Bhutan; 11 Medical College of Soochow University, Suzhou, Jiangsu, People’s Republic of China; 12 Key Laboratory of National Health and Family Planning Commission on Parasitic Disease Control and Prevention, Jiangsu Provincial Key Laboratory on Parasite and Vector Control Technology, Jiangsu Institute of Parasitic Diseases, Wuxi, Jiangsu, People’s Republic of China; 13 Drug Resistance and Diagnostics, Army Malaria Institute, Brisbane, Australia; 14 The AMI Laboratory, QIMR Berghofer Medical Research Institute, Brisbane, Australia; 15 Faculty of Epidemiology and Population Health, London School of Hygiene and Tropical Medicine, London, United Kingdom; 16 Centre for Tropical Medicine, Nuffield Department of Clinical Medicine, University of Oxford, Oxford, United Kingdom; Johns Hopkins Bloomberg School of Public Health, UNITED STATES

## Abstract

**Background:**

The control and elimination of *Plasmodium vivax* will require a better understanding of its transmission dynamics, through the application of genotyping and population genetics analyses. This paper describes VivaxGEN (http://vivaxgen.menzies.edu.au), a web-based platform that has been developed to support *P*. *vivax* short tandem repeat data sharing and comparative analyses.

**Results:**

The VivaxGEN platform provides a repository for raw data generated by capillary electrophoresis (FSA files), with fragment analysis and standardized allele calling tools. The query system of the platform enables users to filter, select and differentiate samples and alleles based on their specified criteria. Key population genetic analyses are supported including measures of population differentiation (*F*_ST_), expected heterozygosity (*H*_E_), linkage disequilibrium (*I*_A_^S^), neighbor-joining analysis and Principal Coordinate Analysis. Datasets can also be formatted and exported for application in commonly used population genetic software including *GENEPOP*, *Arlequin* and *STRUCTURE*. To date, data from 10 countries, including 5 publicly available data sets have been shared with VivaxGEN.

**Conclusions:**

VivaxGEN is well placed to facilitate regional overviews of *P*. *vivax* transmission dynamics in different endemic settings and capable to be adapted for similar genetic studies of *P*. *falciparum* and other organisms.

## Author summary

The *Plasmodium vivax* malaria parasite inflicts significant morbidity in endemic populations across the globe, but has been overshadowed by the more fatal *P*. *falciparum* parasite. In malaria-endemic regions outside of Africa, the declining prevalence of *P*. *falciparum* is coupled with a proportionate rise in *P*. *vivax*, reflecting the greater refractoriness of *P*. *vivax* to transmission interventions. This worrying trend emphasizes the need for a better understanding of the patterns of *P*. *vivax* transmission and spread within and across borders. Genotyping parasite population samples at short tandem repeat (STR) markers such as microsatellites informs on diversity, population structure and underlying transmission patterns. We have established vivaxGEN, an online platform providing a repository for *P*. *vivax* STR genotyping data, and tools for standard population genetic analyses. The platform currently holds publicly available data from 5 vivax-endemic countries that can be browsed on the website (http://vivaxgen.menzies.edu.au). VivaxGEN will support researchers to conduct local STR-based *P*. *vivax* studies with greater autonomy and foster collaborative studies enabling regional overviews of *P*. *vivax* diversity in different endemic settings and across borders. The system can be adapted for STR-based analyses in other microorganisms and the open access source code is provided to facilitate these developments.

## Introduction

In the Asia-Pacific region, *Plasmodium vivax* is responsible for between 20 and 280 million malaria cases per year, inflicting a significant burden of morbidity and mortality. Over the last decade, the prevalence of *P*. *falciparum* has declined in many endemic countries as a result of intensified malaria control interventions, but outside Africa this has been associated with a rise in the proportion of *P*. *vivax* cases, reflecting the limited efficacy of interventions against this species [1]. This trend emphasizes the need for innovative new strategies to reduce *P*. *vivax* transmission. A critical weakness of conventional malaria surveillance is the lack of information on the genetic dynamics of the parasite population—an important reflection of underlying transmission potential. Previous studies have demonstrated the utility of genotyping parasite population samples at highly polymorphic short tandem repeat (STR) markers such as microsatellites to inform on *P*. *vivax* diversity, population structure and underlying transmission patterns [2–19]. These simple molecular approaches complement the more traditional measures of transmission intensity as well as providing a surrogate marker for transmission intensity, informing on outbreak dynamics, reservoirs of infection, and the spread of infection spread within and across borders [20,21].

However, individual projects have limited potential to address regional questions. The challenges of imported and border malaria associated with highly mobile human populations emphasizes the need for a framework to support integrated, multinational comparative analyses. Effective comparison between studies and sites has been confounded by heterogeneity of methodologies such as the number and location of markers used, size standards, allele calling/binning, and specifications for calling minor alleles reflecting minor clones in polyclonal infections [22]. To address some of these challenges, the Vivax Working Group (VxWG) of the Asia Pacific Malaria Elimination Network (APMEN) has worked with research partners in 15 Asia Pacific countries to develop a consensus panel of STR markers (MS1, MS5, MS8, MS10, MS12, MS16, MS20, pv3.27 and msp1F3) and genotyping methods [23]. The web-based VivaxGEN platform was developed to facilitate standardized allele calling, data analysis and sharing across *P*. *vivax* studies using consensus STR marker sets such as the APMEN panel. The VivaxGEN platform provides a repository for FSA files (the primary data files containing the raw fragment analysis data generated during capillary electrophoresis runs). To date, no such repository exists for *P*. *vivax* STR data. The capacity to derive allelic data directly from the FSA files ensures high accuracy and standardization in allele-calling between different sample batches produced at different time points and/or on different machines from possibly different studies. This feature also supports flexibility in defining allele-calling thresholds, enabling user-defined settings that may be applied to one or more sample batches. The VivaxGEN platform also provides tools for standard population genetic analyses that can be applied to multiple sample batches to evaluate local and regional trends in the prevalence of polyclonal infections, population diversity, structure and differentiation both spatially and temporally. Data export tools are available to allow users to conduct more bespoke analyses not provided within the platform framework.

## Methods

### Ethics statement

All genotyping data described in the manuscript has been published [4,9,12,14,34]. As described in the original publications, all samples were collected with written informed consent from the patient, parent or legal guardian (individuals < 18 years of age). Approval was provided by the Institutional Review Board of Jiangsu Institute of Parasitic Diseases (IRB00004221), Wuxi, China; the Research Ethics Board of Health, Ministry of Health Bhutan (REBH 2012/031); the Korea Centers for Disease Control and Prevention Institutional Review Board, Republic of Korea (Protocol No. 2011-02CON-14-P); the Eijkman Institute Research Ethics Commission, Indonesia (EIREC 45/2011); the Ethics Review Board of Addis Ababa University College of Natural Sciences, Ethiopia (RERC/002/05/2013); the Ethics Review Board of Armauer Hansen Research Institute, Addis Ababa, Ethiopia (AHRI-ALERT P011/10); the National Research Ethics Review Committee of Ethiopia (Ref.no. 3.10/580/06); and the Human Research Ethics Committee of the Northern Territory Department of Health and Menzies School of Health Research, Darwin, Australia (HREC 2012–1871, HREC-2012-1895 and HREC-13-1942).

### System architecture and implementation

The VivaxGEN platform was developed as a multi-tier web application system, utilizing PostgreSQL as its backend Relational Database Management System (RDBMS) and leveraging on several common external tools for genotype data analysis. PostgreSQL was chosen as the RDBMS as it provided ACID operations and complex SQL query optimization in an open-source package. The backend is programmed in Python, while the web interface uses JavaScript and jQuery library for interactivity. YAML was chosen as the format for platform configuration and data exchange/interoperability. Sample and assay data uploading process can be performed using either batch processing with tab or comma-delimited files in conjunction with a zip file containing raw FSA files, or interactively using sample and assay editing interface. Detailed instructions on data upload, and an accompanying tutorial dataset can be found in Tutorial 1 (Uploading your metadata and FSA files) provided on the VivaxGEN website and in S1 File.

### Integrated fragment analysis tools

**Fig 1:**
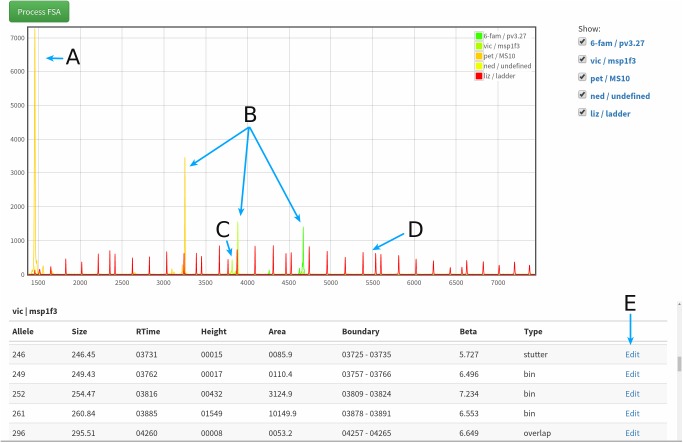
Screenshot from the VivaxGEN platform illustrating the trace viewer features for visual inspection of allele peaks and manual editing of allele annotations. The top panel of the screenshot presents a trace image highlighting examples of a short artefact peak from PET-labelled primer-dimer (A), authentic alleles for each of PET, VIC and 6-FAM-labelled amplicons (B), a stutter peak from the 6-FAM-labelled amplicon (C), and peaks for the LIZ600 size standard (D). The bottom panel of the figure presents the detailed annotation provided for each peak detected by the fragment analysis scan with examples for the VIC-labelled (msp1F3) peaks. The manual edit options (E) whereby the user can change peak annotation details are highlighted.

VivaxGEN provides a framework to store and process raw FSA files with standardized allele calling tools. This framework reduces the heterogeneity that may be introduced from different fragment analysis methods. A Python based library called FATOOLS, which can also be used as a stand-alone command line utility, was developed to provide the raw FSA processing capabilities in VivaxGEN. This library utilizes *numpy* (www.numpy.org) and *scipy* (https://www.scipy.org) scientific libraries for its numerical processing. The library provides methods for base normalization of traces, peak scanning and classification, standard size determination, peak calling and allele annotation, as well as FSA assay quality controls. A detailed guide on the FSA fragment analysis process in VivaxGEN can be found in the Guide on Fragment Analysis manual provided on the website and in S2 File. Briefly, base normalization is undertaken using a *top-hat* morphological transform algorithm implemented in *scipy*. A simple peak finding algorithm and a CWT-based peak scanning algorithm implemented in *scipy* are also included in the library [24]. A combination of greedy algorithm and dynamic programming is employed for standard size alignment and size determination. Results of each step of the FSA and fragment analysis processing are stored in the system for aiding manual inspection and assay verification. The source code for FATOOLS is available for stand-alone usage and further development (http://github.com/trmznt/fatools). To aid the manual inspection of traces, a trace viewer is included in the web interface, as shown in Fig 1. Detailed instructions on the manual data editing tools can be found in Tutorial 2 (Inspecting FSA files and data cleaning) provided on the VivaxGEN website and in S1 File. The trace viewer is coded in JavaScript and enables users to identify and edit incorrectly annotated alleles.

### Tools for allele and sample filtering

The form-based web interface also provides a number of allele and sample filtering options. Details on the allele and sample filtering tools can be found in Tutorial 3 (Data analysis) provided on the VivaxGEN website and in S1 File. Alleles can be filtered according to marker name (Marker), marker failure rate in the given sample set (Marker quality threshold), absolute minimum relative fluorescence unit (RFU) (Allele absolute threshold) and relative RFU of minor peaks compared to the highest intensity peak (Allele relative threshold). Suspected stutter peaks can also be filtered according to a user-defined stutter range in base pairs (Stutter range) and ratio (Stutter ratio) based on the RFU relative to the highest intensity peak in the given range. Samples can also be filtered according to genotyping success rate across the given marker set (Sample quality threshold), to exclude polyclonal infections or multi-locus genotypes that are presented more than once in the given sample set (Sample filtering), or by passive versus active case detection (Detection differentiation).

### Sample query system

Sample querying and grouping can be performed using a query syntax modeled on the NCBI Entrez system with some modification. Detailed instructions on how to perform data analysis using custom queries is provided in Tutorial 4 (Data analysis with custom query) provided on the VivaxGEN website and in S1 File. Boolean operations can be applied to classify sample groups based on spatial (by country level or by 1^st^, 2^nd^, 3^rd^ or 4^th^ administrative division level) or temporal (by year or quartile of sample collection) definitions. The query from the form-based web interface is converted into a YAML-based query internally, which can then be run in the database. An interface that accepts YAML-based query is also provided, enabling the user to apply bespoke sample grouping operations not supported by the form-based web interface. Instructions on how to perform data analysis in VivaxGEN using YAML queries is provided in Tutorial 5 (Data analysis with YAML query) provided on the website and in S1 File.

### Population genetic tools

A suite of population genetic measures and associated statistical tests that are commonly used in STR-based *P*. *vivax* studies to gauge underlying patterns of transmission intensity, stability and boundaries, including rates of polyclonality, population diversity, genetic relatedness, population structure and out-crossing/inbreeding rates, can be applied to the genotyping data from one or more sample batches. Population genetic measures currently supported within VivaxGEN include (i) expected heterozygosity (*H*_E_), an index of population-level diversity, (ii) individual infection and population average measures of the Multiplicity of Infection (MOI), a measure of the genetic complexity within infections, (iii) proportion of polyclonal infections, and (iv) Principal Coordinate Analysis (PCoA) with plots illustrating the population structure and genetic relatedness between infections based on a genetic distance matrix. External software employed by the platform include (i) *LIAN* for measuring linkage disequilibrium (LD) using the index of association (*I*_A_^S^) [25] as a gauge of out-crossing/inbreeding rates, (ii) *Arlequin* for measures of genetic differentiation between populations using the fixation index (*F*_ST_) [26], (iii) the *APE* (Analysis of Phylogenetics and Evolution) package in *R* for building neighbor-joining trees for assessment of genetic relatedness between infections [27], (iv) the *FactoMineR* package in *R* for generating Multiple Correspondence Analysis (MCA) plots to assess population structure and genetic relatedness based on the nominal categorical data [28], and (v) the *DEMEtics* package in *R* for calculating the genetic differentiation index *D* [29,30]. A standardized measure of genetic differentiation, *F*'_ST_, adjusted for marker diversity to support greater comparability between studies using different marker sets is calculated internally in VivaxGEN using the output from *Arlequin* and following the method described by Hedrick [31]. Further details on the population genetic tools can be found in the Guide on Data Analysis manual provided on the VivaxGEN website and in S2 File.

### File format conversion module

The VivaxGEN platform has tools for exporting genotype data in several formats supported by other commonly used population genetics softwares including *LIAN* [25], *Arlequin* [26], *Genepop* [32] and *STRUCTURE* [33]. Tab-delimited formats suitable for R’s data frame or Python’s *pandas* data frame are also provided.

### Data access policy

VivaxGEN users may choose to keep their data private, accessible to all or only specified researchers or they may allow their data to be open access. The repository currently holds data obtained from published studies on *P*. *vivax* samples from China [12], Ethiopia [4], Indonesia [14], South Korea [9] and Bhutan [34]. Private accounts have been generated for users with data sets on *P*. *vivax* samples from Iran, Malaysia, Myanmar, and Vanuatu.

### Availability

The platform can be accessed at http://vivaxgen.menzies.edu.au. The source code for the platform, licensed under GNU GPL version 3, can be obtained from https://github.com/trmznt/plasmogen.

## Results and discussion

**Fig 2:**
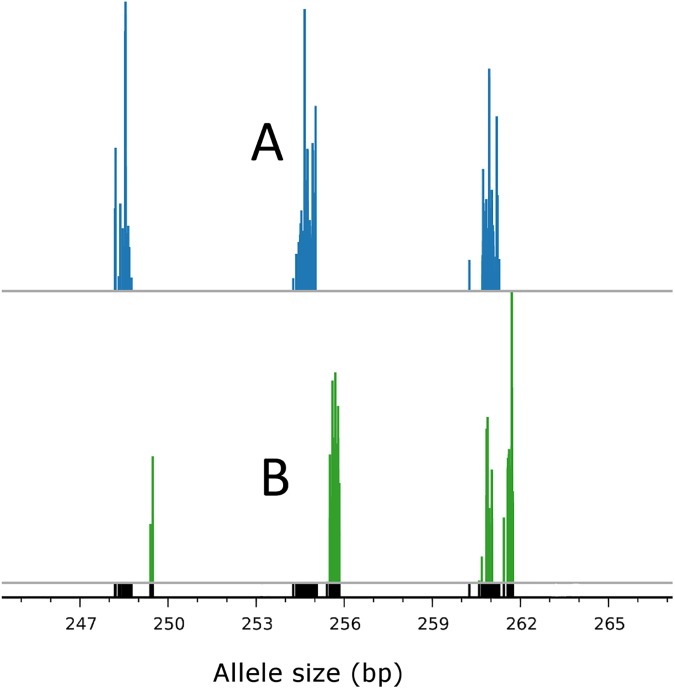
Partial allele summary plot illustrating allele binning. The figure provides a zoomed in view of an allele summary plot presenting MSP1F3 alleles from an Indonesian sample batch (blue allele peaks) and an Ethiopian sample batch (green allele peaks), which were produced by different institutes on different machines. The black allele peaks at the base of the plot are a composite of both the Indonesian and Ethiopian alleles. The allele lengths in the bin defined as allele “256” (i.e. approximating 256 bp) were slightly shifted between Indonesia (A) and Ethiopia (B), highlighting the potential for the same alleles to be assigned to different bins in Indonesia (“255”) versus Ethiopia (“256”). The standardized binning within the VivaxGEN platform ensured that the ~255 bp alleles in Indonesia and the ~256 bp alleles in Ethiopia were assigned to the same allele bin defined as “256”.

**Fig 3:**
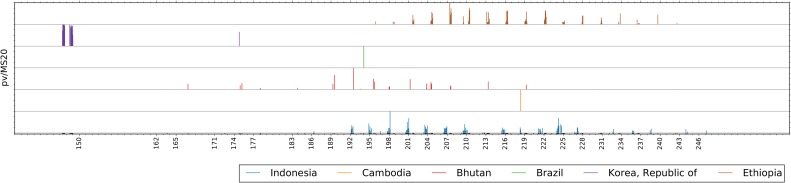
Allele summary plot example for the MS20 locus in samples from different countries. The allele summary plot demonstrates the distinct, population-specific allele profile at the MS20 locus in locally acquired infections from South Korea (purple) versus imported infections from Brazil (green) and Cambodia (orange), and infections from studies conducted in Indonesia (blue), Ethiopia (brown) and Bhutan (red). The plot uses data from VivaxGEN version 1.0.

The VivaxGEN platform was developed as a framework to support standardized allele calling and greater ease of data sharing for comparative analyses between different STR-based studies in *P*. *vivax*. Relative to Single Nucleotide Polymorphisms (SNPs), where a maximum of 4 alleles arising from the 4 different nucleotides are possible at a given position, STRs may exhibit dozens of alleles, measured as different repeat lengths. Although STRs offer high discriminatory potential between independent infections, comparison of STR alleles (fragment size variants) between different sample batches produced at different time points and/or in different laboratories is considerably more challenging than comparison of the discrete allele forms generated from the analysis of SNPs. Despite the application of a size standard, replicates of the same sample may exhibit slight variation (usually less than 1 bp difference) in fragment size. In order to address this variation, alleles can be assigned to bins encompassing a range of fragment sizes usually reflecting the size of the repeat unit. However, whilst one researcher might assign fragment sizes of 254.4 bp and 255.7 bp to two different allele bins such as “254” and “256” respectively, another researcher might assign both alleles to bin “255”, and yet another might assign these fragment sizes to allele bin “256”, creating artificial differentiation between datasets. As illustrated in Fig 2, the VivaxGEN platform provides a common interface for fragment size allele calling using the raw FSA files and applying a standardized binning system, which facilitates comparability between different datasets. By virtue of this feature, using the VivaxGEN platform, it was possible to identify a distinct, population-specific allele profile at the MS20 locus in South Korea versus Bhutan, Ethiopia and Indonesia (Fig 3). The distinct MS20 allele profile observed in South Korea is postulated to reflect a single major reservoir of *P*. *vivax* infections, most likely from North Korea [9]. Future data entries to VivaxGEN on MS20 genotypes from other vivax-endemic regions are likely to provide further important insights on this phenomenon and other transmission patterns.

One of the greatest challenges in genotyping *Plasmodium* samples (and other microorganisms) is the identification and characterization of polyclonal infections [22]. Owing to artefacts such as background noise, stutter peaks, and overlapping peaks (also known as pull-up peaks or bleed) in multiplex reactions where amplicons are labelled with different fluoresceins. Some of these artefacts may not be automatically detected and excluded from the peak binning during the fragment scanning process. To address this challenge, the VivaxGEN platform provides utilities enabling visual inspection of individual electropherogram traces and editing of allele annotations. The platform also enables user-defined relative minimum RFU thresholds for calling minor alleles: an approach that is commonly applied in STR-based *Plasmodium* studies to reduce the prevalence of artefact peaks, and enhance comparability in the sensitivity to detect minor peaks in samples of differing quality such as DNA derived from dried blood spots versus blood tubes [35]. Different studies may however apply different thresholds. A benefit of the integrated database and analytical framework in VivaxGEN is that population genetic measures such as the average MOI or proportion of polyclonal infections can be compared between different sample batches at the same user-defined threshold–and indeed multiple different thresholds can be explored.

**Fig 4:**
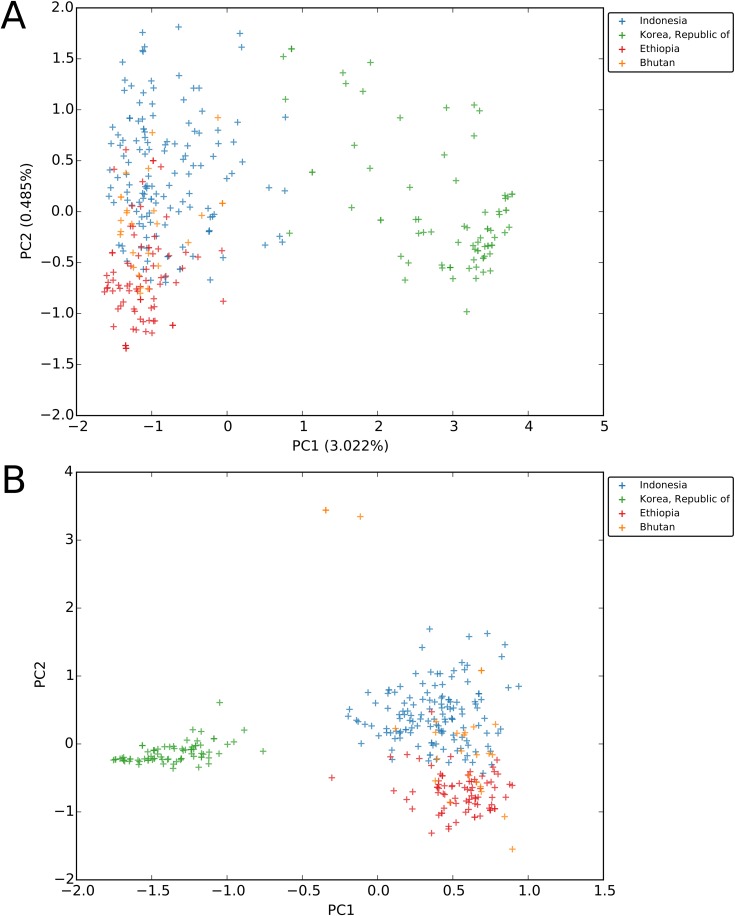
**MCA plots using 9 APMEN markers (panel A) and 5 APMEN markers (panel B) between Bhutan, Ethiopia, Indonesia and South Korea samples.** The plot uses data from VivaxGEN version 1.0.

Capitalizing on the feature to incorporate samples from multiple studies (batches) within an analytical procedure, we used the platform to compare multi-locus genotypes (MLGs) between different published datasets stored in the database. As illustrated in Fig 4A, Multiple Correspondence Analysis (MCA) demonstrated clear distinction of the MLGs at the 9 APMEN standard markers between Ethiopia, Indonesia and South Korea, whilst the Bhutanese isolates displayed a broad range of MLGs with overlap in both Ethiopia and Indonesia. It is widely acknowledged that different STR markers have different strengths in their ability to detect polyclonal infections and/or to define population structure [36]. Amongst the APMEN panel, 5 markers (MS1, MS5, MS10, MS12 and MS20) have been defined as “stable”, with optimal utility for analysis of population differentiation [36]. Therefore, the effect of repeating the analysis using the 5 stable markers was assessed (Fig 4B). A similar pattern was observed to the full marker panel, adding assurance that the clustering patterns had not been affected by the high diversity markers.

The integrated data repository, allele calling and data analysis tools in VivaxGEN promote exploratory and semi-interactive analysis in a common web interface. Compared to other popular softwares for processing microsatellite data, VivaxGEN is unique in providing both the capability to process and store raw electropherogram data (FSA files) and to perform statistical and population genetic analysis commonly applied in studies of *Plasmodium* (Table 1). A data export utility enables population genetic analysis outputs for a given parameter set to be downloaded from VivaxGEN to facilitate data reporting. These features greatly simplify data processing and exploration, and should enable malaria researchers who are new to the field of population genetics to conduct robust data analysis with greater autonomy. The integrated data repository should also foster collaborations between different research institutions and allow analyses on regional trends as well as population differences between countries. The outcomes will inform national malaria control and elimination programs on malaria transmission dynamics, may help distinguish local from imported parasite populations and facilitate malaria surveillance.

**Table 1:** Comparison of functionality between several software/platforms for microsatellite data processing.

Software/Platform	Raw electrophoregram processing	Fully automatic binning	Cross-platform	Interoperability with common population genetics software
Genemapper	✓	✓	✕	✕
Peakscanner	✓	✕	✕	✕
Allelogram	✕	✓	✓	✕
Flexibin	✕	✓	✓	✕
Allelobin	✕	✓	✓	✕
TANDEM	✕	✓	✓	✓ (export file)
MICROSATELIGHT	✕	✓	✓	✕
VivaxGEN	✓	✓	✓	✕

## Conclusions

The VivaxGEN platform is well placed to facilitate regional overviews of *P*. *vivax* population genetic patterns in different endemic settings, informing on the underlying transmission dynamics of this highly adaptive parasite. The system is amenable to being adapted for STR-based analyses in *P*. *falciparum* and other microorganisms or other forms of genetic data such as SNP-based genotypes. The open access source code is provided to facilitate developments for such applications.

## Supporting information

S1 File VivaxGEN tutorials. (PDF)

S2 File VivaxGEN user guide. (PDF)
