# Supplementary material for: VivaxGEN: An open access platform for comparative analysis of short tandem repeat genotyping data in Plasmodium vivax populations
Source: PLoS Negl Trop Dis. 2017 Mar 31;11(3):e0005465. doi: 10.1371/journal.pntd.0005465 (PMC5389845; doi:10.1371/journal.pntd.0005465)
Supplement: S1 File — (PDF) [file pntd.0005465.s001.pdf]

VivaxGEN Tutorial

Release

Hidayat Trimarsanto, Sarah Auburn

Oct 13, 2016

1 UPLOADING YOUR DATA 1
1.1 Preparing Input Files . . . 1
1.2 Creating a New Batch . . . 2
1.3 Adding Sample Data . . . 2
1.4 Adding FSA Zip File . . . 3
1.5 Processing FSA Microsatellite Data . . . 3
1.6 Assessing Fragment Analysis Results . . . 3

2 MICROSATELLITE DATA CLEANING 5
2.1 Inspecting Peaks . . . 5
2.2 Inspecting Traces and Re-annotating Peaks . . . 7
2.3 Inspecting Alleles with Lower Absolute Threshold or Relative Threshold . . . 9

3 DATA ANALYSIS 11
3.1 Inspecting Sample Metadata . . . 11
3.2 PCoA Cluster Plot . . . 13
3.3 PCoA Cluster Plot with Sample Grouping by Spatial Differentiation . . . 14
3.4 MCA Cluster Plot with Multiple Batches . . . 15

4 DATA ANALYSIS WITH CUSTOM QUERY 17
4.1 Using the Custom Query Form . . . 17
4.2 Performing Principal Coordinate Analysis using a Custom Query . . . 19

5 DATA ANALYSIS WITH YAML FORMAT 21
5.1 A Glance of YAML Format . . . 21
5.2 Using YAML Query . . . 22
5.3 Using YAML Query for Differentiation . . . 22

UPLOADING YOUR DATA

Contents

- *UPLOADING YOUR DATA*
  - *Preparing Input Files*
  - *Creating a New Batch*
  - *Adding Sample Data*
  - *Adding FSA Zip File*
  - *Processing FSA Microsatellite Data*
  - *Assessing Fragment Analysis Results*

This tutorial provides step-by-step directions on how to prepare and upload your FSA files and metadata to the VivaxGEN platform, with an accompanying example *Plasmodium vivax* **microsatellite** dataset. A more detailed guide for data preparation and uploading can be found in the Guide: Data preparation and Uploading. Note, it is assumed that users have a good understanding of the general concepts of microsatellite-based genotyping.

1.1 Preparing Input Files

The example datasets, available as data-01.zip, consist of three files:

1. `sampleinfo.txt` - A tab-delimited file containing sample metadata.
2. `fsa.zip` - A zipped file containing all microsatellite data in FSA-formatted files.
3. `assayinfo.txt` - A tab-delimited file containing FSA metadata

The `sampleinfo.txt` and `assayinfo.txt` files can be opened for inspection using any software capable of reading text files or spreadsheet-based softwares such as Microsoft Excel or LibreOffice Calc.

More detailed information on the file format can be found here: Guide - File Format.

When preparing your own input files, any name can be used for each of the three files, but the file formatting must be strictly adhered to.

Please note that the system is primarily set up to accept data from *Plasmodium vivax*. If data is available for other *Plasmodium Spp*, this can be accommodated using the same input file formatting as long as the relevant fields are filled appropriately. *It is strongly recommended to use different batches for different species* as downstream analytical processes will require separation of the different species.

1.2 Creating a New Batch

Before uploading the example datasets, you will need to create a new batch (unless you plan to add data to an existing batch). A batch is essentially a collection of samples and associated molecular data which the user intends to analyse together (i.e. from the same study).

Log in to VivaxGEN using the guest or private account details. To establish a private account, with private username and passowrd, you will need to send an email request to the systems administrator at anto@eijkman.go.id.

Once logged in to VivaxGEN, select **Manage data** or **Browse >> Batch** from the navigation menu. A list of existing batch names, including publically available batches, will be displayed (these names cannot be used for new batches). To create a new batch, select **New batch**. To add data or update an existing batch, you will need to select the intended batch name instead.

For new batches, you will be provided with a form with details to fill in as listed below. Compulsory fields are stated.

Batch code *Compulsory field.* A unique (i.e. not already present in the database) string that identifies your batch. Allowed characters for the string are alphanumerics, dash or underscore. The maximum length for batch code is 16 characters. **Do not use any spaces**. Best practise is to use a combination of country identification, species and year, such as IDPV2015 for Indonesian *P vivax* in year 2015.

Description *Optional but recommended field.* A brief description outlining the nature of the samples and the study for which the data was generated.

Primary group *Compulsory field.* A string indicating the name of the group or organization providing the data, i.e. the data owner. For this tutorial, set as DEMOGROUP.

Assay provider group *Compulsory field.* A string indicating the name of the organization where the assays were run. For the accompanying example datasets in this tutorial, set as MACROGEN.

Batch for bins setting *Compulsory field.* This is the batch code that will be used as the reference for bin settings. This option allows different batches to have different bins settings and parameters (for examples, bins for LIZ600 and bins for LIZ500). For now, just use **default**.

Species *Compulsory field.* A string indicating the Plasmodium Spp. The system currently supports Pv and Pf assays. For this tutorial, set as **Pv**. *Important*: by setting species, the system will assume that any markers without explicit species code mentioned in any input files are markers for this intended species, unless the species is explicitly stated. For example, marker **MS16** will be assumed as **pv/MS16**.

Remarks *Optional field.* An optional field for any information regarding this batches, further detailed description on the samples or the study that may be helpful for those who are going to use the data in this batch.

Once you have completed the forms, select **Save**. You will then be directed to the batch view page, where you can manage the given batch.

1.3 Adding Sample Data

On the Batch view, select **Choose file** or **Browse** next to **Sample Info** file, and select the sample info file (sampleinfo.tab in the tutorial). Then select **Upload** to temporarily save the sample information file in the VivaxGEN platform. Select **Verify** to check if the sample information file contains any errors. In case of errors, a message detailing the error lines will be returned. Correct any errors and re-upload the sample information file. Ensure that the appropriate sample submission option is checked - for the tutorial, leave as default (Add new samples and update existing samples). Select **Proceed** to save the sample information file in the VivaxGEN platform. On the **Uploading Report** view, if the sample information was uploaded successfully, select **Continue** to return to the Batch view.

1.4 Adding FSA Zip File

From the Batch view, under **FSA Bulk Uploading**, select **Start** upload session. In the FSA Bulk Upload Manager view, click on **Select and upload FSA archive file** and select the fsa zip file (fsa.zip in the tutorial) to upload the FSA files to VivaxGEN. Once the uploading is finished, select **Continue to verify the uploaded archive file** to check that the files were uploaded correctly. In case of errors, a message detailing the error lines will be returned. Correct any errors and re-upload the fsa zip file.

If there are no errors, click on **Continue to upload FSA info file (CSV or tab-delimited)** and select the assay information file (assayinfo.tab in the tutorial) to the VivaxGEN platform. Select **Continue to verify FSA info file** to check that the file was uploaded correctly. In case of errors, a message detailing the error lines will be returned. Correct any errors and re-upload the assayinfo file by selecting **Change/replace the uploaded FSA info file**.

If there are no errors, select **Continue to process FSA files** to save each of the FSA files to the VivaxGEN platform. This process may take a few minutes. Once uploading is finished, select **Continue** to return to the Batch view. In case FSA Bulk Uploading is interrupted at any point, you can return to the incomplete session by selecting **List pending sessions** and then selecting the corresponding session.

1.5 Processing FSA Microsatellite Data

Once the FSA files have been saved, *fragment analysis* (see the manuscript for further details on this process) must be undertaken. From the Batch view, select **Start FSA FA Manager**, and then select **Process FSA**. Note that this is a lengthy task, with the time required depending on the number of FSA files/assays submitted, and how "noisy" the traces are. Please also note that you can continue other tasks in VivaxGEN in parallel or log out of the platform without impeding the fragment analysis processing. If you choose to log out during this processing step, on returning to VivaxGEN, you can navigate back to the FSA FA manager view to inspect progress. Once assay processing is finshed, select **Continue**, and then select **Browse FSA** files to starting inspecting individual FSA files as described in step 6 (or batch name to return to the batch view). Note that further filtering of alleles by absolute and relative allele peak intensity are provided in the analysis tools.

1.6 Assessing Fragment Analysis Results

Once the fragment analysis process has finished, it is recommended to assess the results of the processing. On the FSA FA manager view, select **Browse FSA** files to open a new page showing the list of the uploaded FSA files together with their parameter results.

The details of the parameters are outlined below:

FSA Filename The name of the FSA file

Sample Code The sample code for the corresponding FSA file

Panel The panel used for the corresponding FSA file

Score The quality of ladder peaks of the FSA file, from 0.00 to 1.00 (highest score).

RSS The Residual Sum of Squares of the ladder peaks against the regression line. Lower RSS value (< 50.00) indicates higher quality of the FSA file.

Proctime The time taken for the system to process the FSA file in miliseconds. Higher processing time usually indicates that the FSA file is noisy.

To inspect individual FSA files, select the corresponding FSA filename (good practise is by right-clicking the mouse button to open a new tab) which will open the FSA viewer. Individual peaks (alleles) can be manually edited (or re-annotated) by selecting the **Edit** link in the corresponding peak/allele tables. Once the allele-calling has been finalized, several population genetic analyses can be performed using a suite of tools available under **Analyze** in the navigation menu (see Tutorial 2). Note that further filtering of alleles by absolute and relative allele peak intensity thresholds are provided in the analysis tools.

If there are any errors at any of steps in the process that cannot be resolved, please contact the systems administrator at anto@eijkman.go.id.

TUTORIAL 2

MICROSATELLITE DATA CLEANING

Contents

- *MICROSATELLITE DATA CLEANING*
  - *Inspecting Peaks*
  - *Inspecting Traces and Re-annotating Peaks*
  - *Inspecting Alleles with Lower Absolute Threshold or Relative Threshold*

This tutorial provides step-by-step instructions on how to perform data cleaning to exclude peaks such background noise, artefacts or stutter. The fragment analysis process in VivaxGEN provides the first layer of data cleaning from the raw FSA files. Several algorithms, described further in the *Integrated Fragment Analysis Tools* section of the manuscript, perform automated annotation of peaks as either bin peaks (real alleles) or non-binned peaks such as stutter and artefacts, which are excluded from further data analysis. However, some peaks may not be annotated correctly by the algorithms, especially in challenging FSA files such as those containing low intensity peaks or extensive background noise. The VivaxGEN platform therefore provides tools for inspecting the raw electropherogram traces to cross-check the automated annotations, and to manually re-annotate peak definitions where needed. As the tutorial requires uncleaned data, you will need to use the new sample batch that you created and processed in Tutorial 1.

2.1 Inspecting Peaks

We will start by inspecting the summary of all genotyped peaks (i.e. all peaks defined as true alleles by the automated fragment analysis algorithms) in the data set using the Genotype Summary tool. Select the **Genotype Summary** entry from the **Analyze** drop-down menu. This will take you to a web page with a form for selecting the sample batch and markers, and for sample and marker filtering according to a number of parameters described further in the *Tools for Allele and Sample Filtering* section of the manuscript. A snapshot of the form is provided in the figure below.

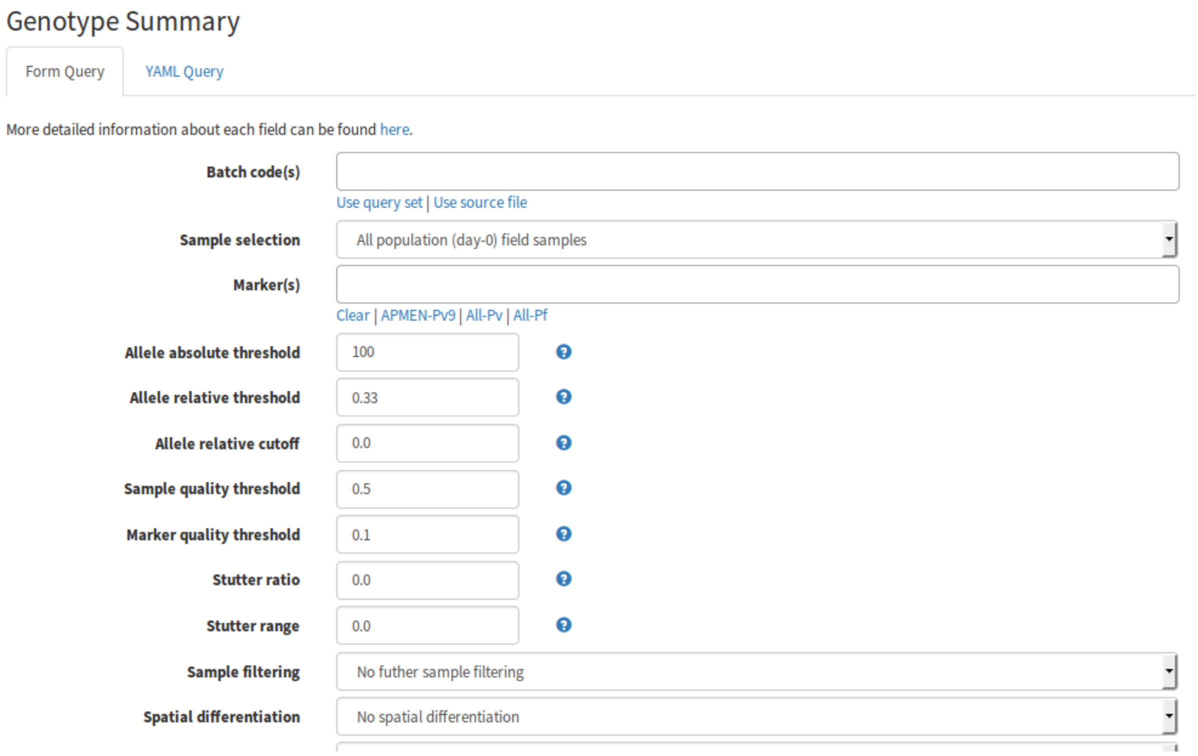

In the **Batch code(s)** field, select the new, uncleaned batch that you created in Tutorial 1. The samples in the Tutorial 1 batch were genotyped at the 9 APMEN *P. vivax* markers (see reference) - click on the **APMEN-Pv9** link to select these markers. This will populate the **Marker(s)** field with the appropriate markers. In this step of the tutorial, we will leave all other parameters as default. Note that the default value of the **Allele absolute threshold** is set to 100 relative fluorescence units (RFU) i.e. all peaks with RFU less than 100 will be excluded even if they were binned by the automated algorithm. Select **Execute** to perform the analysis. Once complete, a *Genotype Summary* report will be provided as illustrated in the figure below.

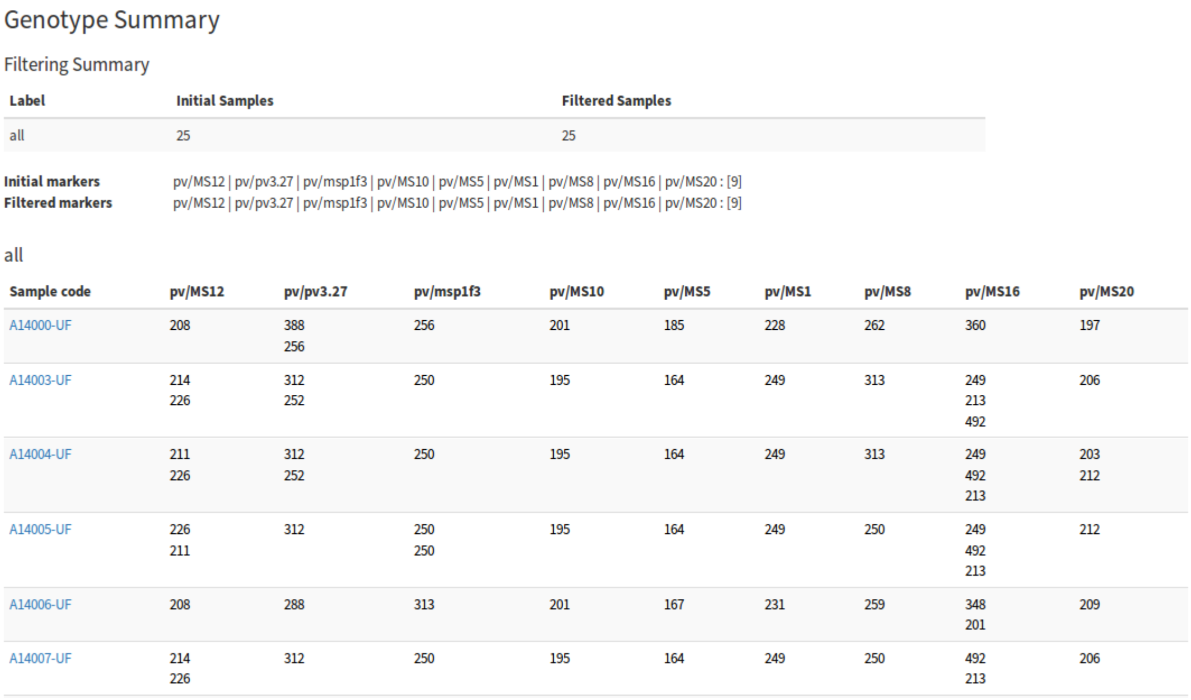
Genotype Summary

Filtering Summary

Label	Initial Samples	Filtered Samples
all	25	25

**Initial markers**	pv/MS12 | pv/pv3.27 | pv/msp1f3 | pv/MS10 | pv/MS5 | pv/MS1 | pv/MS8 | pv/MS16 | pv/MS20 : [9]
**Filtered markers**	pv/MS12 | pv/pv3.27 | pv/msp1f3 | pv/MS10 | pv/MS5 | pv/MS1 | pv/MS8 | pv/MS16 | pv/MS20 : [9]

all

Sample code	pv/MS12	pv/pv3.27	pv/msp1f3	pv/MS10	pv/MS5	pv/MS1	pv/MS8	pv/MS16	pv/MS20
A14000-UF	208	388<br>256	256	201	185	228	262	360	197
A14003-UF	214<br>226	312<br>252	250	195	164	249	313	249<br>213<br>492	206
A14004-UF	211<br>226	312<br>252	250	195	164	249	313	249<br>492<br>213	203<br>212
A14005-UF	226<br>211	312	250<br>250	195	164	249	250	249<br>492<br>213	212
A14006-UF	208	288	313	201	167	231	259	348<br>201	209
A14007-UF	214<br>226	312	250	195	164	249	250	492<br>213	206

If we look closely at the results, we will see that there are sample/marker combinations that have multiple alleles. For example, there are multiple alleles in many of the samples at markers **MS12** and **MS16**. The multiple alleles within sample/marker combinations are sorted by their heights. In some cases, all of the alleles may be real, reflecting multiple clone infections in which different clones have different alleles. However, in some cases, one

or more of the alleles may reflect peaks from noise, stutter, overlap or other artefacts that were not correctly annotated by the automated fragment analysis algorithms. *Please note that the VivaxGEN platform is under constant development to improve features such as the fragment analysis peak annotation and the results of the automated fragment analysis peak annotation may differ slightly between different versions of the platformwhen this tutorial was written.*

2.2 Inspecting Traces and Re-annotating Peaks

To aid judgment of whether a given peak reflects a true allele, we need to inspect the original electropherogram trace in the FSA file. We can do this using the FSA Viewer, which can be accessed directly by clicking on an allele of interest.

UFA-01-MN	223	304	262	174	197<br>224	216<br>234	214	369	224<br>203
UFA-02-MN	208	316	262	222	185	228	280	342	197<br>224

As an example, locate sample UFA-01-MN as indicated above, and open the trace view for allele 224 of MS5 marker by clicking on 224 using the right mouse button and selecting open link in new tab. The trace view should look similar to the figure below.

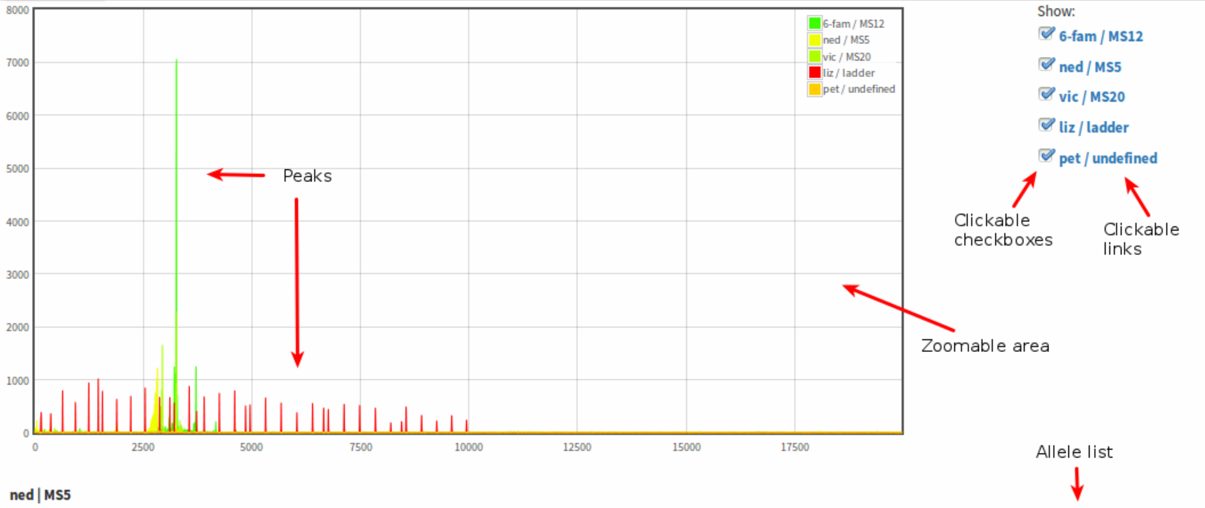

ned | MS5

Allele	Size	RTime	Height	Area	Boundary	Beta	Type	
[illegible]	[illegible]	[illegible]	[illegible]	[illegible]	[illegible]	[illegible]	[illegible]	Edit
194	194.12	02777	00748	6586.5	02769 - 02784	8.806	stutter	Edit
197	197.12	02827	01222	10107.3	02820 - 02834	8.271	bin	Edit
221	221.46	03237	00060	0563.1	03230 - 03247	9.384	stutter	Edit
224	222.91	03262	00943	3016.0	03259 - 03264	3.198	bin	Edit

The view consists of the electropherogram trace from the FSA file and a summary of allele peaks and associated annotations below. We can zoom in on the trace by selecting an area using the mouse. To the right of the trace is a panel containing checkboxes which allow the user to turn specific markers/dyes on or off. Clicking on the dye or marker name will scroll the allele list to the designated dye/marker.

As an example, turn off all dyes/markers except MS5 and zoom in on the trace to the area around 2400 - 3400 retention time to generate a view similar to the figure below.

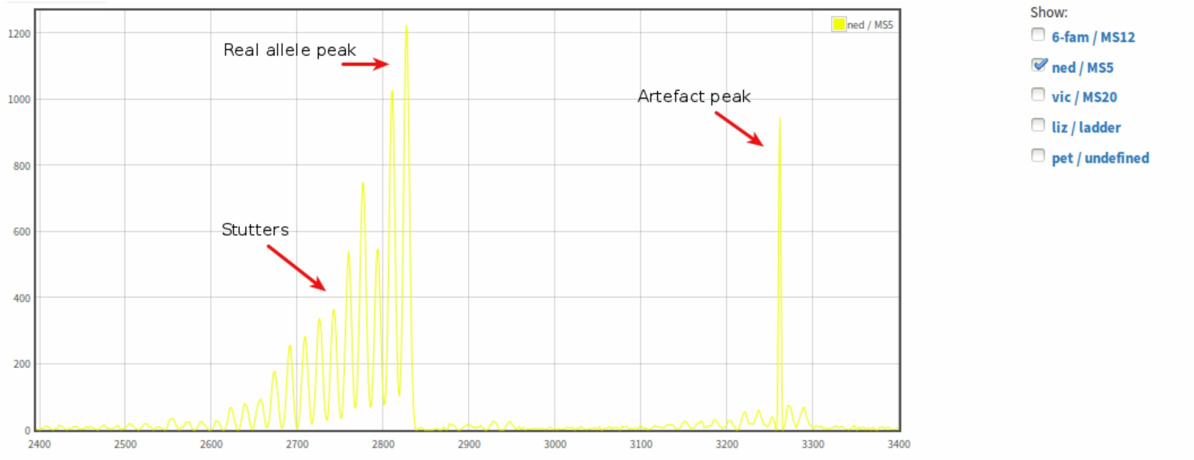

ned | MS5

Allele	Size	RTime	Height	Area	Boundary	Beta	Type	
197	197.12	02827	01222	10107.3	02820 - 02834	8.271	bin	Edit
221	221.46	03237	00060	0563.1	03230 - 03247	9.384	stutter	Edit
224	222.91	03262	00943	3016.0	03259 - 03264	3.198	bin	Edit

Note that the peak underlying allele 224 at retention time ~3262 has a very narrow base uncharacteristic of the alleles for this marker. The *beta* value (height divided by width) of this peak is 3.2, whereas most real alleles have beta of 5-10. Moreover, the peak is not accompanied by small stutter peaks or widening areas close to baseline that usually form around real allele peaks. If we switch the other markers (**MS12** and **MS20**) back on and zoom back into the region, we can see that the MS5 224 allele is overlapped by a large MS12 peak and is therefore likely to be an overlap peak and not a true allele (as illustrated below).

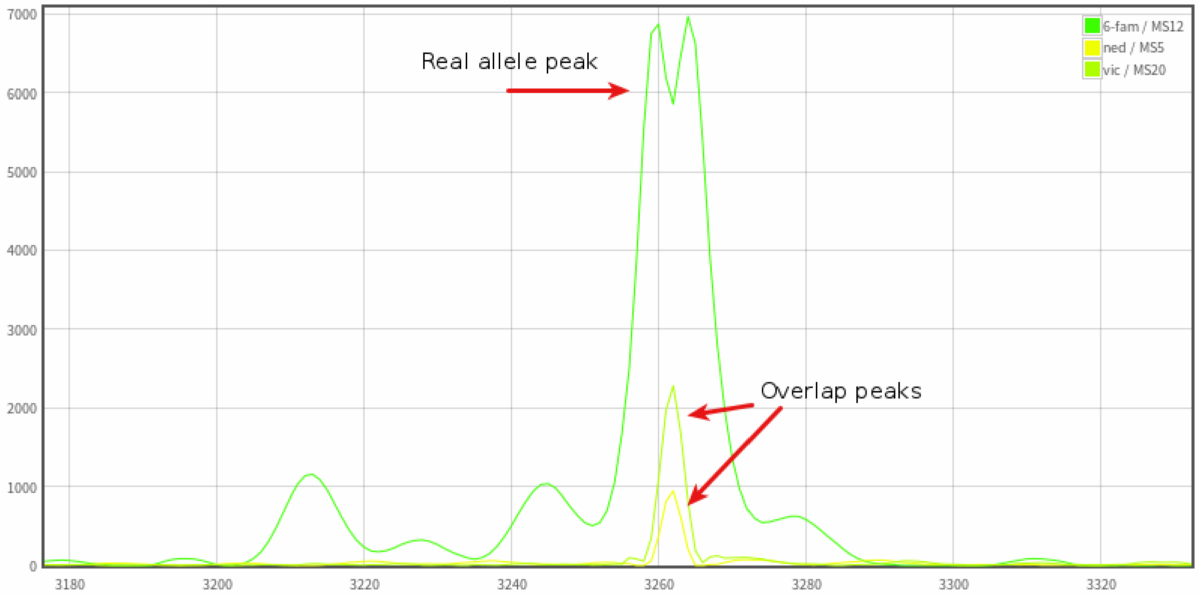

We can manually re-annotate this peak as overlap peak (or any other annotation other than bin) to exclude it from further analyses. To do so, click on the **Edit** link at the row of allele 224, which will bring up a pop up window as illustrated below.

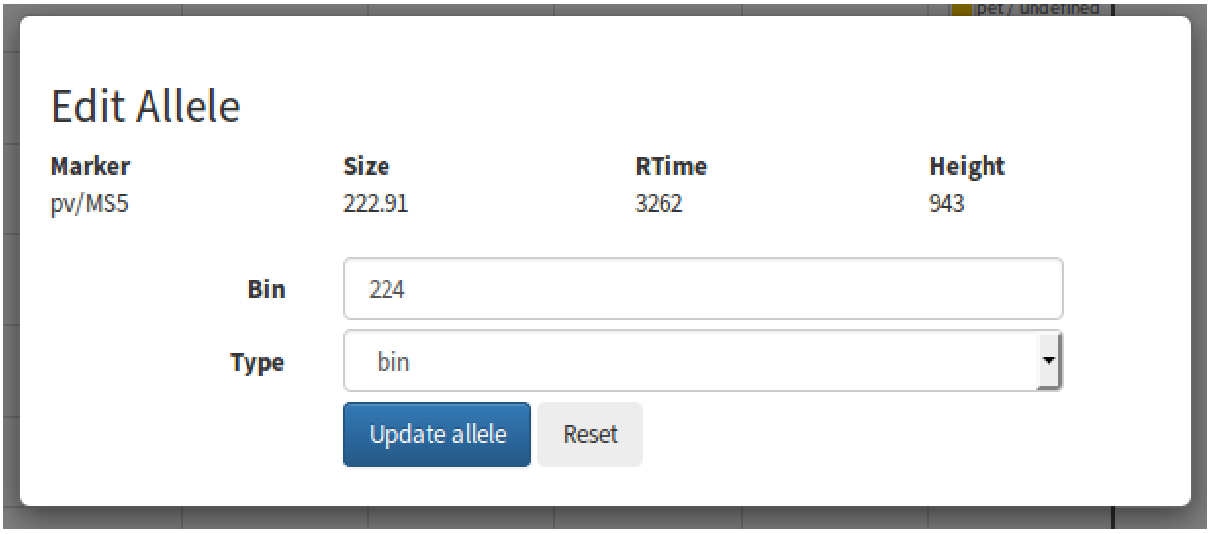

From the pop-up window, we can change **Type** field from bin to overlap (or any other annotation other than bin) and then click **Update allele**. The updated annotation will be saved. We can then return to the Genotype Summary tab (or window), and click on **Resubmit analysis** on the top left of the page to get the updated result. The allele 224 at MS5 marker from sample UFA-01-MN should not appear in the updated Genotype Summary.

2.3 Inspecting Alleles with Lower Absolute Threshold or Relative Threshold

In many cases, false allele peaks may be excluded by adjusting the **Allele absolute threshold** and/or **Allele relative threshold**. In the previous steps, we observed our data at the default **Allele absolute threshold** of 100 RFU. To inspect the Genotype Summary at a lower threshold, we can change the value of the **Allele absolute threshold** and/or **Allele relative threshold** to lower values in the Genotype Summary form. Note that the public batches available in VivaxGEN have all been cleaned to **Allele absolute threshold** of 40 RFU.

TUTORIAL 3

DATA ANALYSIS

Contents

- *DATA ANALYSIS*
  - *Inspecting Sample Metadata*
  - *PCoA Cluster Plot*
  - *PCoA Cluster Plot with Sample Grouping by Spatial Differentiation*
  - *MCA Cluster Plot with Multiple Batches*

This tutorial provides step-by-step instructions on how to perform data analyses using the form-based web interface. The tutorial uses examples from the publically available batch BTPV, comprising microsatellite data on *P. vivax* isolates from patients in Bhutan (1).

3.1 Inspecting Sample Metadata

Before performing further analyses on batch BTPV, we will start by determining how many samples the batch has, and inspecting some features of the sample metadata. For this step, we will use the *Sample summary* analysis tool.

Select the **Sample summary** entry from the **Analyze** drop-down menu, and a form similar to the figure below will appear.

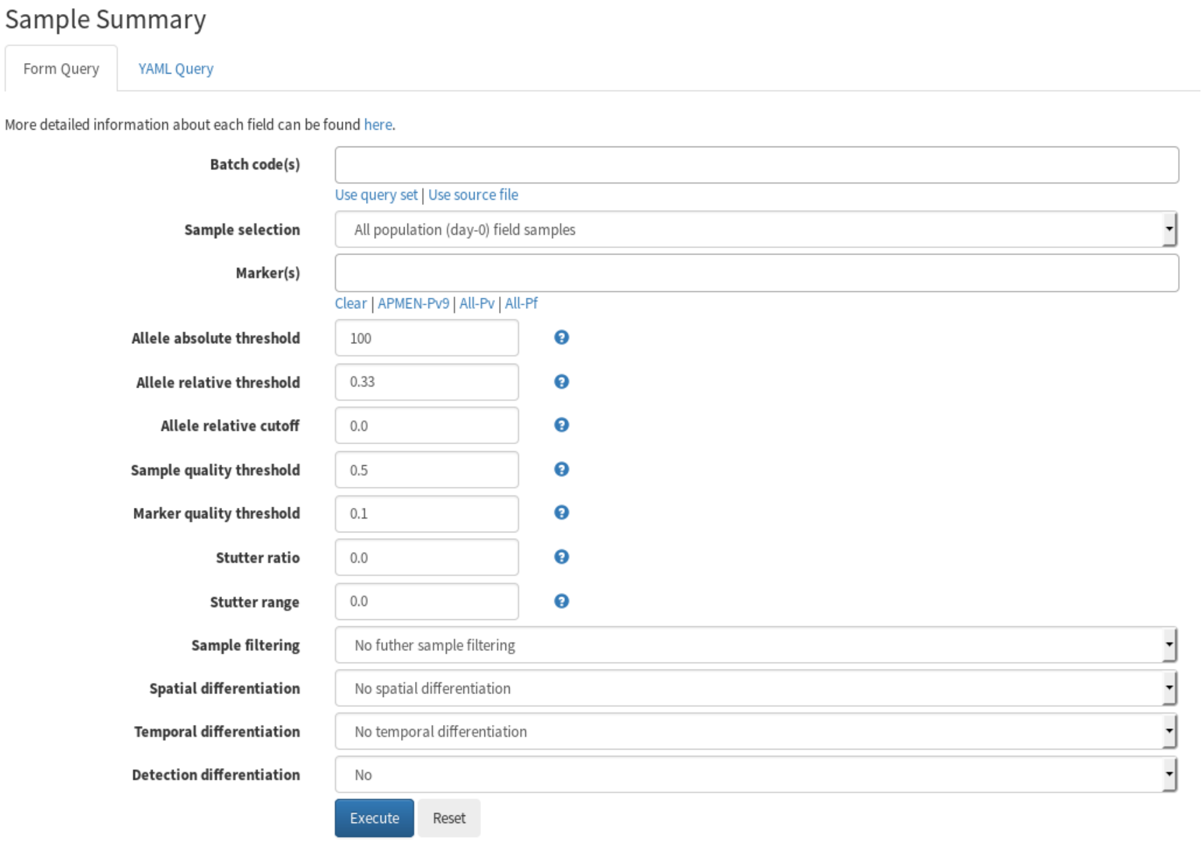

To use the BTPV batch, select BTPV from the **Batch code(s)** field. As we plan to analyze all independent samples in the batch (i.e. all day-0 samples), the **Sample selection** field should be left as default. The samples in the BTPV batch were genotyped at the 9 APMEN P. vivax markers (2), click on the **APMEN-Pv9** link to select these markers. This will populate the **Marker(s)** field with the appropriate markers.

As detailed in the *Tools for Allele and Sample Filtering* section of the manuscript, samples and markers can be filtered according to a number of parameters. In this tutorial, we will use all of the default parameters. Once the parameters have been set, select **Execute** to perform the analysis. A snapshot of the report outlining the results of the sample summary query on the BTPV batch is illustrated in the figure below.

Sample/Metadata Summary Report

Filtering Summary

Label	Initial Samples	Filtered Samples
all	28	28

**Initial markers**	pv/MS12 | pv/pv3.27 | pv/msp1f3 | pv/MS10 | pv/MS5 | pv/MS1 | pv/MS8 | pv/MS16 | pv/MS20 : [9]
**Filtered markers**	pv/MS12 | pv/pv3.27 | pv/msp1f3 | pv/MS10 | pv/MS5 | pv/MS1 | pv/MS8 | pv/MS16 | pv/MS20 : [9]

all

string1	N
	28

passive_detection	N
True	28

symptomatic_status	N
False	28

pcr_identity	N
pv	28

microscopy_identity	N
pv	28

blood_withdrawal	N
venous	28

imported_case	N
	28

The following information is provided:

- There are 28 samples in total (Initial Samples) in batch BTPV.
- After filtering samples and markers according to the parameters set in the prior step, there are 28 samples remaining (Filtered Samples) in batch BTPV and that have been included in the analysis. As defined in the default parameters, the dataset on the 28 Filtered samples only comprises samples with genotype calls for at least 50% of the total markers (i.e. 5 of the 9 markers), as the default value of **Sample quality threshold** is 0.5. As the default **Allele absolute threshold** was set to 100, genotype calls were only provided for alleles with relative fluorescence unit (RFU) >= 100.
- All 28 Filtered samples were collected by passive detection, without any symptomatic status, and had been identified as *P. vivax* by both PCR and microscopy. All blood samples were collected by venous withdrawal.
- The patient donors included 16 Indian nationals and 12 Bhutanese nationals. Three of the patients were female and 25 were male.

3.2 PCoA Cluster Plot

In this tutorial step, we will review the steps for performing Principal Coordinate Analysis (PCoA) in the BTPV batch as an example on how to apply one of the platform's suite of standard population genetic tools using the form-based web interface.

Select the **Principle Coordinate Analysis (PCoA)** entry from the **Analyze** drop-down menu. A form similar to that used for inspecting the sample metadata will be provided to enable sample and marker filtering as required. Note that the sample and marker filtering form is available for all analyses within the VivaxGEN platform.

Fill the form as before:

- Use **Batch code(s)** BTPV
- Use **Markers(s)** APMEN-Pv9
- Leave all other fields as default

When the form has been filled, select **Execute** to run the analysis. The results output should provide a PCoA cluster plot similar to the figure below. This is the simplest form of analysis, with minimal annotation by metadata details such as spatial and temporal parameters.

Principal Coordinate Analysis (PCoA) Result

Filtering Summary

Label	Initial Samples	Filtered Samples	MLG Samples
all	28	28	28

Initial markers pv/MS12 | pv/pv3.27 | pv/msp1f3 | pv/MS10 | pv/MS5 | pv/MS1 | pv/MS8 | pv/MS16 | pv/MS20 : [9]
Filtered markers pv/MS12 | pv/pv3.27 | pv/msp1f3 | pv/MS10 | pv/MS5 | pv/MS1 | pv/MS8 | pv/MS16 | pv/MS20 : [9]

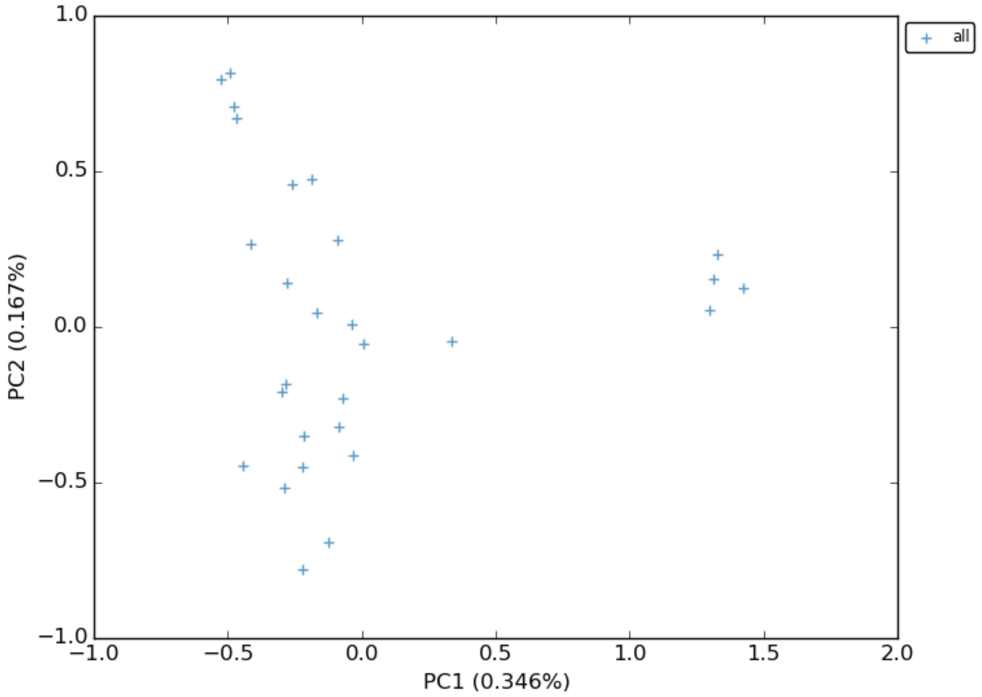

Note that PCoA can only be performed on samples with genotype calls for all markers selected (i.e. with complete multi-locus genotypes - MLGs). The Filtering Summary in the results output provides sample numbers for Initial Samples, Filtered Samples, and samples with complete MLGs (MLG Samples).

3.3 PCoA Cluster Plot with Sample Grouping by Spatial Differentiation

In this tutorial step, we will further explore the PCoA cluster plot in batch BTPV by overlaying more details on spatial differentiation. Return to the Principle Coordinate Analysis (PCoA) entry and use the same parameters as in step 2 except for the **Spatial differentiation** field – here, select *1st Administration level*. When the form has been filled, select **Execute** to run the analysis. As illustrated in the figure below, a similar cluster plot to step 2 should be produced but with annotation on spatial differentiation at the 1st Administrative level. With the extra colour-coded spatial annotation, we can now see that the 4 samples which separated from the others on PC1 were all collected from Sarpang District.

Label	Initial Samples	Filtered Samples	MLG Samples
all | bhutan / gelephu	1	1	1
all | bhutan / pemagatshel	1	1	1
all | bhutan / samdrupjongkhar	1	1	1
all | bhutan / sarpang	16	16	16
all | bhutan / trongsa	2	2	2
all | bhutan / tsirang	1	1	1
all | bhutan / wangdiphodrang	6	6	6

**Initial markers**	pv/MS12 | pv/pv3.27 | pv/msp1f3 | pv/MS10 | pv/MS5 | pv/MS1 | pv/MS8 | pv/MS16 | pv/MS20 : [9]
**Filtered markers**	pv/MS12 | pv/pv3.27 | pv/msp1f3 | pv/MS10 | pv/MS5 | pv/MS1 | pv/MS8 | pv/MS16 | pv/MS20 : [9]

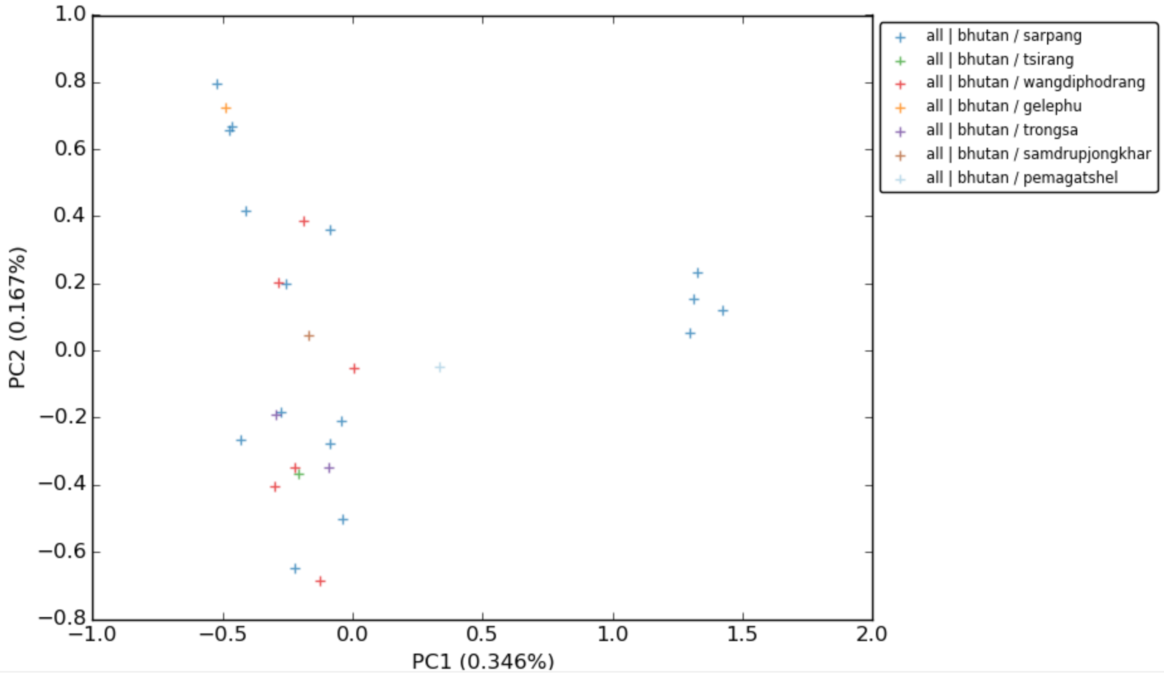

Note that the Filtering Summary now provides details on sample numbers by 1st Administration level.

3.4 MCA Cluster Plot with Multiple Batches

A more interesting question that can be inferred by cluster plot is comparing samples from different bigger regions such as countries, which can be done by using multiple batches from different countries. In this tutorial step, we will use Multiple Correspondence Analysis (MCA) which is another method of getting cluster plot.

Select the Multiple Correspondence Analysis (MCA) entry from the **Analyze** drop-down menu. A familiar form will be shown. However, instead of just selecting BTPV batch in the **Batch code(s)** field, add another batch by selecting ETPV batch which contains samples from Ethiopia. We also need to select *Country level* for **Spatial differentiation** field so that we will know which samples come which countries. The completed form will look similar to the following figure.

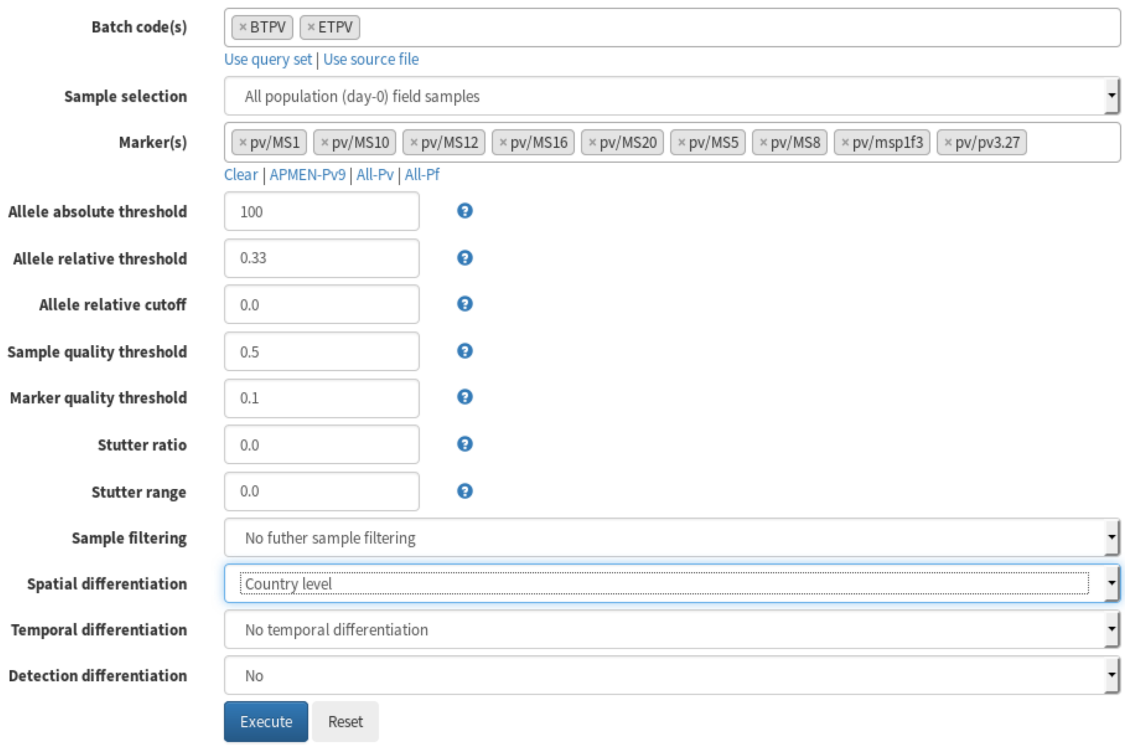

The reason we are confident in mixing BTPV and ETPV is that both batches were genotyped using APMEN 9 *P. vivax* markers, and both used LIZ600 standard size.

Once the MCA analysis finishes, we will obtain a plot similar to the following figure.

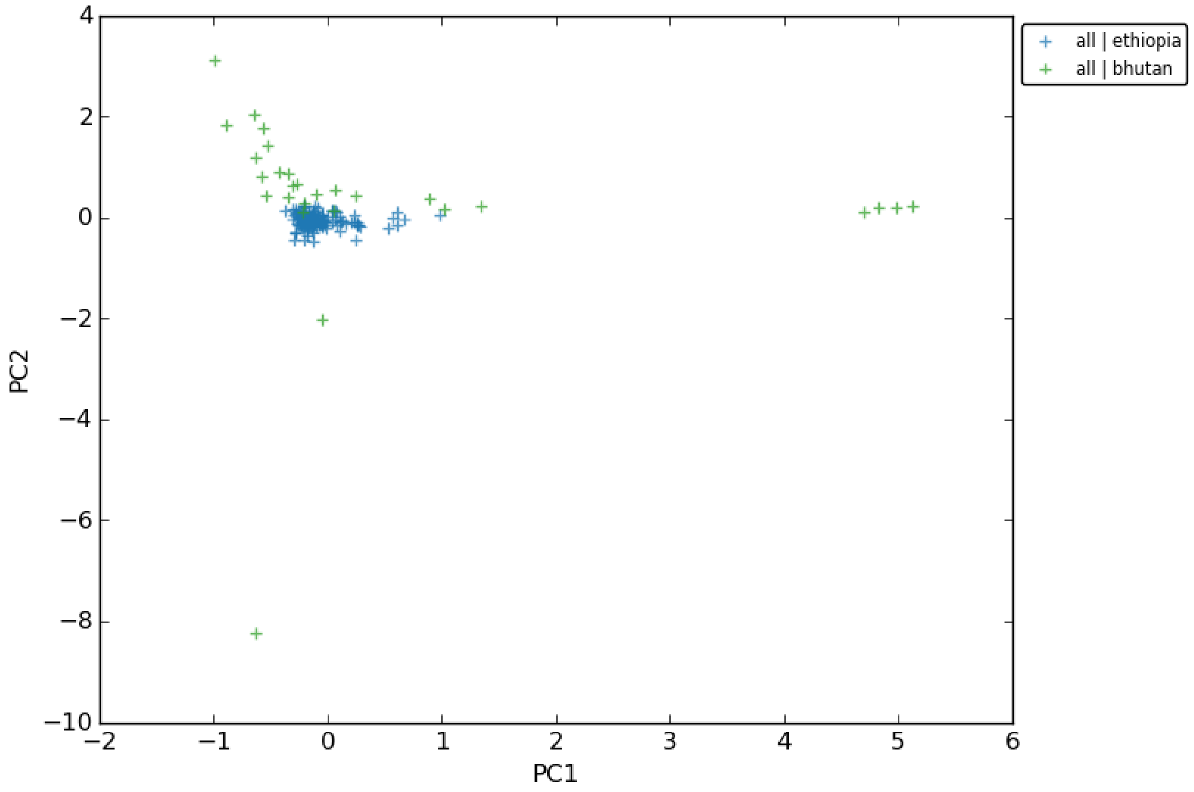

We can see from the result that the sample size from Ethiopia is much bigger than from Bhutan. Despite of this, the Bhutan cluster is more dispersed compared to the the much tight Ethiopia cluster. In molecular term, we can infer that the Bhutan samples have higher diversity relative to the Ethiopia samples. Please note that there are other methods that can be used to check and confirm these diversities, such as **Heterozygosity** analysis.

TUTORIAL 4

DATA ANALYSIS WITH CUSTOM QUERY

Contents

- *DATA ANALYSIS WITH CUSTOM QUERY*
  - *Using the Custom Query Form*
  - *Performing Principal Coordinate Analysis using a Custom Query*

In this tutorial, we will review the steps for performing data analysis using the custom query tools. The custom query tools may be required for analyses that cannot be performed using the existing drop-down options in the form-based query tools available on VivaxGEN. For example, if we are interested in only using samples from certain geographical areas that cannot be selected using the form-based web tools discussed in Tutorial 3. The custom query tools in VivaxGEN are modelled on the NCBI Entrez system, which uses the following syntax:

```
value[FIELDNAME]
```

For demonstration purposes, we will use the BTPV batch again for this tutorial. Recall from Tutorial 3 that the samples in the BTPV batch have the following values in the **nationality** field: india and bhutan. We will review the custom query steps to differentiate the BTPV samples by the nationality field in Principal Coordinate Analysis (PCoA).

4.1 Using the Custom Query Form

As a start, let's review the BTPV sample summary after differentiation by the nationality field. Select the **Sample summary** entry from the **Analyze** drop-down menu. Next, instead of selecting the batch code as in Tutorial 3, click on the Use query set link just below the **Batch code(s)** field. The **Batch code(s)** field will change to a **Query set** field as illustrated in the following figure.

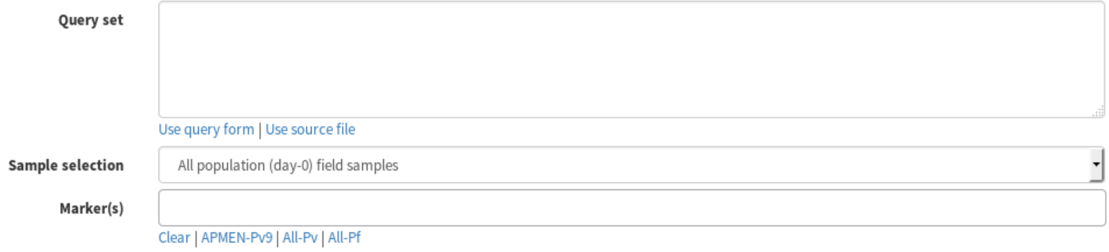

Write or copy the query statement below into the **Query set** field:

```
BTPV[batch] !! india[nationality] >> India $ bhutan[nationality] >> Bhutan
```

Note, in simple terms, the above query statement essentially makes the following commands:

- use samples from the BTPV batch: "`BTPV[batch]`""

- create a sample set labelled *India* from the samples that have value *india* in the **nationality** field: “`india[nationality] >> India`“
- create another sample set labelled *Bhutan* from the samples that have bhutan in the nationality field: “`bhutan[nationality] >> Bhutan`“
- the double exclamation symbol `!!` indicates that the former statement will apply to all sample set, so in this case all the sample set must come from BTPV batch
- the dollar sign `$` is the sample set separator
- the >> sign indicates the label string

In summary, the above query statement performs re-grouping of the samples in batch BTPV by nationality, enabling comparison of samples from Indian versus Bhutanese nationals.

After adding the query statement, as in Tutorial 3, select the **APMEN-Pv9** marker set in the **Marker(s)** field, and leave the other parameters as default as illustrated in the figure below. Select **Execute** to perform the analysis.

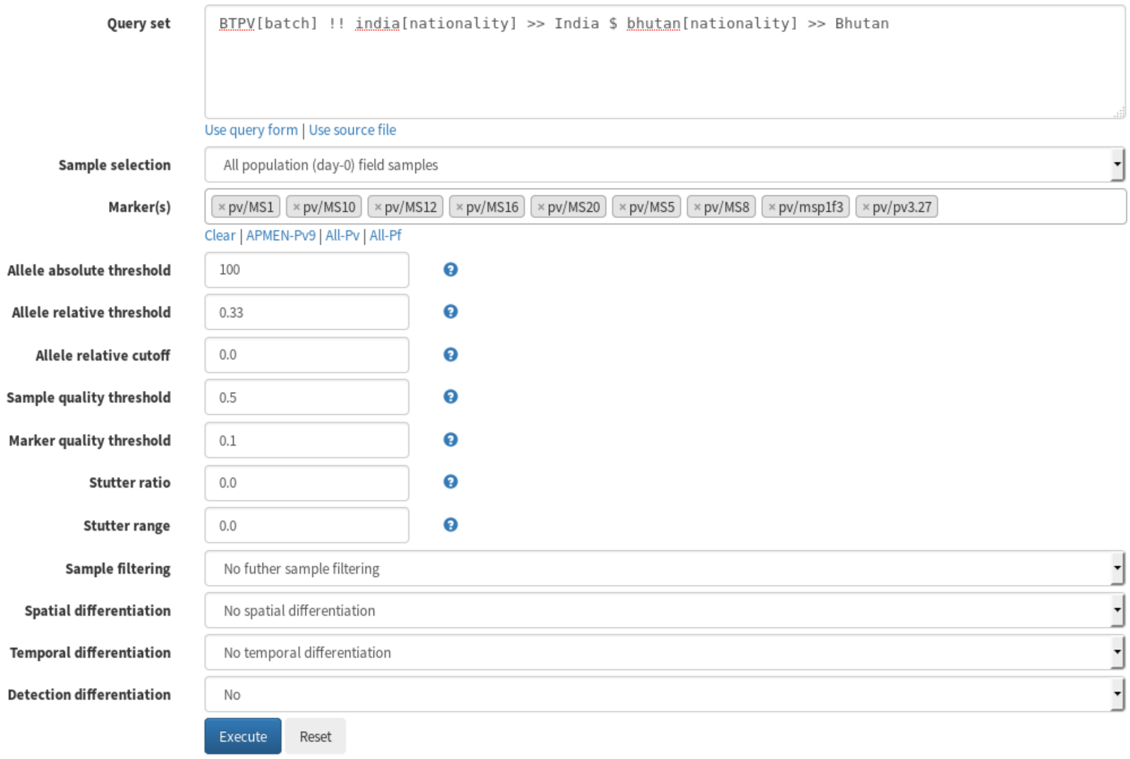

A snapshot of the report outlining the results of the customized sample summary query is illustrated in the figure below.

Filtering Summary

Label	Initial Samples	Filtered Samples
Bhutan	12	12
India	16	16

**Initial markers**	pv/MS12 | pv/pv3.27 | pv/msp1f3 | pv/MS10 | pv/MS5 | pv/MS1 | pv/MS8 | pv/MS16 | pv/MS20 : [9]
**Filtered markers**	pv/MS12 | pv/pv3.27 | pv/msp1f3 | pv/MS10 | pv/MS5 | pv/MS1 | pv/MS8 | pv/MS16 | pv/MS20 : [9]

Bhutan

string1	N
	12

passive_detection	N
True	12

symptomatic_status	N
False	12

pcr_identity	N
pv	12

microscopy_identity	N
pv	12

blood_withdrawal	N
venous	12

imported_case	N
	12

As defined in the custom query statement, the results are reported for each of the Indian and Bhutanese nationality sample sets, labelled *India* and *Bhutan* respectively.

4.2 Performing Principal Coordinate Analysis using a Custom Query

In this step of the tutorial, we will generate a PCoA cluster plot on the BTPV batch with differentiation by nationality. Select the **Principle Coordinate Analysis (PCoA)** entry from the **Analyze** drop-down menu. As in step 1, click on the **Use query set** link, and add the query statement from step 1. Select the **APMEN-Pv9** marker set, leave the other parameters as default, and then select Execute.

The results output should provide a PCoA cluster plot with colour-coded differentiation of the samples by nationality as in the figure below.

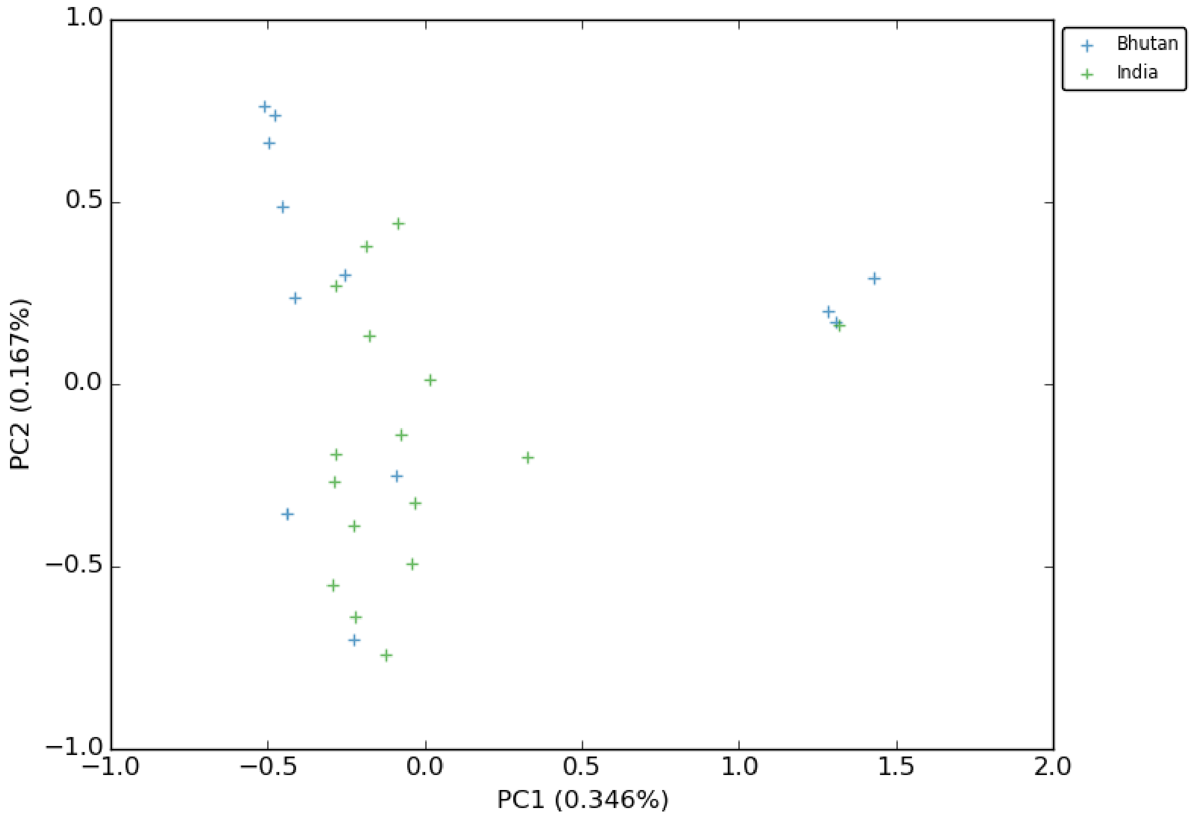
Bhutan
India
1.0
0.5
0.0
−0.5
−1.0
PC2 (0.167%)
−1.0
−0.5
0.0
0.5
1.0
1.5
2.0
PC1 (0.346%)

TUTORIAL 5

DATA ANALYSIS WITH YAML FORMAT

Contents

- *DATA ANALYSIS WITH YAML FORMAT*
  - *A Glance of YAML Format*
  - *Using YAML Query*
  - *Using YAML Query for Differentiation*

In this tutorial, we will review options for performing data analysis in VivaxGEN using the YAML text format. Note, most users will not need to use the YAML text format – options are provided for more advanced users performing bulk analyses. As stated in the official YAML website, YAML is *a human friendly data serialization standard for all programming languages*. YAML is a text format that computers can parse and that users can edit and read easily. The YAML format for querying VivaxGEN may be useful to save time where a user needs to perform multiple analyses using different tools with similar queries. In this case, the user can copy and paste the YAML query into the YAML query set, save the query for future use, or for sharing with other users to ensure consistent parameters (and consistent results).

5.1 A Glance of YAML Format

An example of YAML format for VivaxGEN is shown below (please note that the indentation of the text is important):

```
selector:
  Bhutan:
    - { batch: BTPV }

filter:
  markers: [ MS1,MS10,MS12,MS16,MS20,MS5,MS8,msp1f3,pv3.27 ]
  abs_threshold: 50
  rel_threshold: 0.33
  rel_cutoff: 0
  sample_qual_threshold: 0.1
  marker_qual_threshold: 0.1
  sample_option: A
  peak_type: [binned]
  stutter_ratio: 0.5
  stutter_range: 3.5

differentiator:
  spatial: -1
  temporal: -1
```

The `selector` section indicates the sample set that the user wants to create. The above query essentially states that we want to create 1 sample set labelled “Bhutan” which will contain samples from the BTPV batch.

The `filter` section controls the parameters used to perform sample, marker and allele filtering. The options are similar to the form-based web fields.

The `differentiator` section indicates the type of sample differentiation that the user would like to perform on the samples. In the above query, we do not use any differentiation and so we use *-1* for all parameter values.

5.2 Using YAML Query

To use the YAML format as a query, for example to prepare a *Sample summary*, we need to click on the **YAML Query** navigation tab which will change the web form to a simple form as illustrated below.

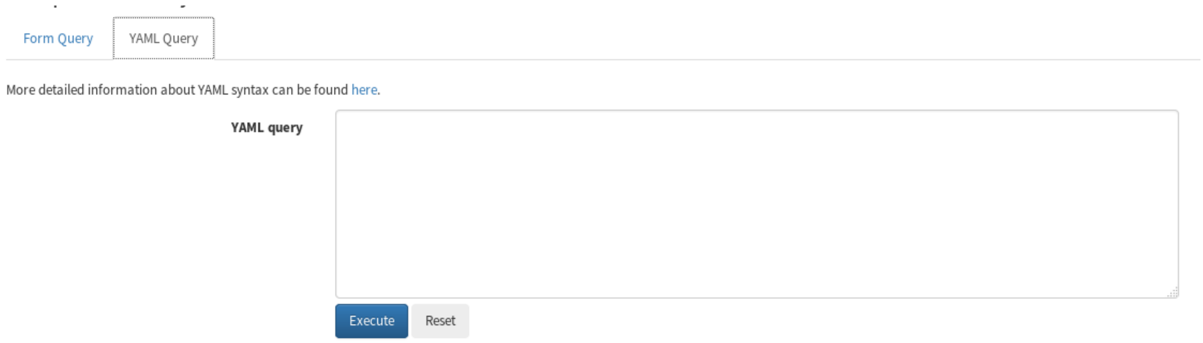

The YAML text then needs to be pasted or directly written into the **YAML query** field.

For the tutorial, select the **Sample summary** entry from the **Analyze** drop-down menu, and paste the above example query into the **YAML query** field. Select **Execute** to perform the analysis. The result output should be similar to the output from section 1 in Tutorial 3.

5.3 Using YAML Query for Differentiation

Next, we will perform the Principal Coordinate Analysis (PCoA) performed in section 4 of Tutorial 3 using the YAML query format. The YAML query text that we need to use is shown below:

```
selector:
  all:
    - { batch: BTPV }
    - { batch: ETPV }

filter:
  markers: [ MS1,MS10,MS12,MS16,MS20,MS5,MS8,msp1f3,pv3.27 ]
  abs_threshold: 50
  rel_threshold: 0.33
  rel_cutoff: 0
  sample_qual_threshold: 0.5
  marker_qual_threshold: 0.1
  sample_option: A
  peak_type: [binned]
  stutter_ratio: 0.5
  stutter_range: 3.5

differentiator:
  spatial: 0
  temporal: -1
```

Note that we have defined a single sample sets labelled *all*, and set `spatial` in `differentiator` with value of `0` for country (use `1` for 1st administrative level, `2` for 2nd administrative level, etc).

Once we execute the above query, we should get the same result as in section 4 of Tutorial 3.

Similary, we can perform the same analysis as in Tutorial 4 i.e. re-grouping the samples by nationality. For this, we need the following query:

```
selector:
  Bhutan:
    - { batch: BTPV, nationality: bhutan }
  India:
    - { batch: BTPV, nationality: india }

filter:
  markers: [ MS1,MS10,MS12,MS16,MS20,MS5,MS8,msp1f3,pv3.27 ]
  abs_threshold: 50
  rel_threshold: 0.33
  rel_cutoff: 0
  sample_qual_threshold: 0.5
  marker_qual_threshold: 0.1
  sample_option: A
  peak_type: [binned]
  stutter_ratio: 0.5
  stutter_range: 3.5

differentiator:
  spatial: -1
  temporal: -1
```

Note that we have defined two sample sets labelled *Bhutan* and *India*, with each sample set having a different nationality value but the same batch code.

If we execute the above query in PCoA tool, we should get similar result as in Tutorial 4. Please note that PCoA and Multiple Correspondence Analysis (MCA) will perform *data jittering* which essentially add small noises to the actual data to prevent to prevent overlap of samples and, hence, different PCoA or MCA plots may look slightly different despite the same data set being applied.
