# Supplementary material for: VivaxGEN: An open access platform for comparative analysis of short tandem repeat genotyping data in Plasmodium vivax populations
Source: PLoS Negl Trop Dis. 2017 Mar 31;11(3):e0005465. doi: 10.1371/journal.pntd.0005465 (PMC5389845; doi:10.1371/journal.pntd.0005465)
Supplement: S2 File — (PDF) [file pntd.0005465.s002.pdf]

VivaxGEN User Guide

Release 1.0

Hidayat Trimarsanto, Sarah Auburn

Oct 13, 2016

1 GUIDE ON DATA ANALYSIS **1**
1.1 SAMPLE PROCESSING 1
1.2 DATA SUMMARIES 2
1.3 POPULATION GENETIC ANALYSES 3
1.4 COMPARATIVE POPULATION GENETIC ANALYSES 3

2 FILE FORMATS **5**
2.1 Sample Information File Format 5
2.2 FSA Information File Format 6
2.3 Controlled Vocabularies / Keywords 7

Bibliography **9**

CHAPTER 1

GUIDE ON DATA ANALYSIS

Contents

- *GUIDE ON DATA ANALYSIS*
  - *SAMPLE PROCESSING*
  - *DATA SUMMARIES*
    - *Sample summary*
    - *Allele summary*
    - *Genotype summary*
  - *POPULATION GENETIC ANALYSES*
    - *Multiplicity of Infection (MoI)*
    - *Expected Heterozygosity*
    - *Linkage Disequilibrium (LD)*
  - *COMPARATIVE POPULATION GENETIC ANALYSES*
    - *Genetic Differentiation using the Fixation Index (F_{ST})*
    - *Jost's D Index*
    - *Principal Coordinate Analysis (PCoA)*
    - *Multiple Correspondence Analysis (MCA)*
    - *Neighbor-Joining Analysis*

This document provides a summary of the analyses which can be performed in VivaxGEN.

1.1 SAMPLE PROCESSING

The sample processing steps enable the user to select **genotype data** for specific **sample sets** and **marker sets**, and to define if/how the samples should be grouped in downstream analyses.

1. Sample selection and grouping

   The platform creates a sample set based on the user query settings by identifying samples in the database with metadata values concordant with the user selection criteria. The samples are then assigned to groups according to the user-defined differentiation settings. Using the user-defined marker set, the platform identifies the corresponding genotype data for each sample in the sample set, and differentiates the genotype data in accordance with the sample differentiation settings.

2. Allele selection and filtering

The platform collects all binned alleles (i.e. called alleles) at the user-defined markers for each sample in the user-defined sample set, and then filters out all alleles that do not meet the user-defined allele absolute threshold, allele relative threshold, allele relative cut-off, stutter ratio and stutter range.

3. Sample quality filtering

   Using the allele-filtered data set the platform determines the proportion of genotyping fails (i.e. proportion of markers with no binned alleles) for each sample. Samples with less than the user-defined proportion of successfully genotyped markers (**Sample quality threshold**) are filtered out of the sample and corresponding genotype data sets.

4. Marker quality filtering

   Using the sample quality-filtered samples and corresponding genotype data sets, the platform determines the proportion of genotyping fails for each marker. Markers with less than the user-defined proportion of successfully genotyped samples (**Marker quality threshold**) are filtered out of the marker and corresponding genotype data sets.

5. Sample characteristics filtering

   Additional user-defined sample filtering options are performed on the data sets remaining after step 4 (i.e. on the sample and marker quality-filtered data sets). Additional sample filtering options include selection of monoclonal samples only. For the monoclonal sample setting, the platform identifies all samples with multiple alleles at one or more of the given markers, and subsequently filters out these samples and their corresponding genotype data. Another sample filtering option is the selection of strict/low-complexity samples. For the strict/low-complexity sample setting, the platform identifies all samples with multiple alleles at two or more one of the given markers, and subsequently filters out these samples and their corresponding genotype data. A further sample filtering option is the selection of unique genotype samples. For the unique genotypes sample setting, the platform identifies and excludes samples with incomplete multi-locus genotypes (MLGs) and then identifies and excludes consecutive samples with identical MLGs and their corresponding genotype data.

6. Filtered sample sets

   After step 5, the genotype data is ready to be analysed.

1.2 DATA SUMMARIES

The following tools provide simple descriptive statistics of a given data set. Utilities of these tools include, but are not limited to, background checks on the sample metadata, and allele and marker summaries, which can be a useful aid in data editing/data annotation.

1.2.1 Sample summary

This tool provides metadata summaries on a given sample set.

1.2.2 Allele summary

This tool provides summaries of the alleles in a given data set, including the number of unique alleles, the number and frequency of successful genotype calls for each allele, and the quality of the allele binning.

1.2.3 Genotype summary

This tool provides an overview of the allele calls in a given data set with the data organized with samples in rows and markers in columns. Where a given sample/marker combination has multiple alleles, each allele is presented in a separate row, with alleles ordered by peak intensity, whereby the predominant allele (highest intensity peak) is on the top row. The genotype summary is a useful aid in data editing/data annotation: the FSA trace for an allele can be easily accessed from this view by simply clicking on the allele of interest.

1.3 POPULATION GENETIC ANALYSES

Depending on the analytical procedure, the platform will either use *all alleles*, *predominant alleles* or *MLGs* (*multi-locus genotypes* comprising the predominant alleles at each locus in samples with complete data only). Several analyses are restricted to the predominant alleles to ensure an unbiased estimate of the minor allele frequency *[Anderson2000]*. Analyses requiring complete data, such as those entailing the construction of distance matrices, are restricted to MLGs.

To avoid potential bias in allele frequency estimates resulting from the inclusion of samples that are not independent, such as pairs of day-0 and recurrent samples, it is advisable to restrict population genetic analyses to day-0/independent samples (defined as "population samples" in VivaxGEN).

1.3.1 Multiplicity of Infection (MoI)

This tool provides statistics on Multiplicity of Infection and the proportion of polyclonal samples by sample group and by marker. Statistics on the significance of the differences between sample groups are provided for the proportion of polyclonal samples.

A sample is defined as polyclonal if any of the given markers have more than one allele. The MOI in each sample is defined by the maximum number of alleles observed at any of the given markers. The MOI provides a lower bound estimate of the number of genetically distinct parasite clones within a sample.

This tool uses all available alleles in each sample.

1.3.2 Expected Heterozygosity

Expected heterozygosity (HE) provides a measure of population diversity at a given marker or averaged across a range of markers for a given sample set. The expected heterozygosity for each marker is calculated using the equation given below, where *pi* is the frequency of the *i* th of *k* alleles.

$$H_E = (\frac{n}{n-1})(1 - \sum_{i=1}^{k} p_i^2)$$

Values range from 0 (no diversity) to nearly 1 (large number of equally frequent alleles). Only the predominant allele at each marker in each sample is used for this analysis.

1.3.3 Linkage Disequilibrium (LD)

Multi-locus linkage disequilibrium (LD) is assessed by the standardised index of association (I_A^s) using LIAN 3.5 software *[Haubold2000]*. Testing the null hypothesis of linkage equilibrium, the significance of the (I_A^s) estimates is assessed using 100,000 random permutations of the data.

Using the additional sample filtering options described in section 1.5, users can derive LD estimates for all samples, strict/low-complexity samples and unique genotypes in a given sample sets. Comparison of the results aids the detection of any recent clonal expansions, whereby the IAS is expected to drop substantially in the unique genotypes relative to the full (all) sample set.

This tool uses MLG samples (requires complete data).

1.4 COMPARATIVE POPULATION GENETIC ANALYSES

1.4.1 Genetic Differentiation using the Fixation Index (F_{ST})

This tool measures the genetic differentiation between sample groups using pairwise measures of the fixation index (F_{ST}), using Arlequin software version 3.5.5.2 *[Excoffier2010]*. In addition to the classic F_{ST}, VivaxGEN

calculates a standardized measure of the genetic differentiation (F'_{ST}), which adjusts for high marker diversity *[Hedrick2005]*. The F'_{ST} provides a measure of F_{ST} expressed as a fraction of the maximum possible value of this statistic, whereby

$$F'_{ST} = \frac{F_{ST}}{F_{STmax}}$$

F_{STmax} is calculated by recoding the data to obtain the maximum divergence among populations.

This tools uses MLG samples.

1.4.2 Jost's D Index

This tool measures the genetic differentiation between sample groups using Jost's D index. Jost's D index incorporates normalization of the genetic data by heterozygosity, thus providing adjustment for high marker diversity *[Jost2008]*. VivaxGEN uses the DEMEtics library from the R statistical suite to perform this analysis.

This tools uses MLG samples

1.4.3 Principal Coordinate Analysis (PCoA)

Principal Coordinate Analysis (PCoA) is a method to generate cluster plots, which are useful to inspect the relatedness (or allele similarity) between samples. The method works by first generating a genetic distance matrix, and then performing PCA (Principal Component Analysis) on the distance matrix. The genetic distance between any two samples is defined as the proportion of differing alleles between their MLGs.

This tools uses MLG samples.

1.4.4 Multiple Correspondence Analysis (MCA)

Multiple Correspondence Analysis (MCA) is another method to generate cluster plots, similar to PCoA. While PCoA uses a genetic distance matrix to measure the genetic distance between samples, MCA uses the allele data directly and treats each allele as a discrete (categorical/nominal) data point. Put in simple terms, MCA is to qualitative data, as PCoA is to quantitative data.

VivaxGEN employs the FactoMineR library from the R statistical software to perform the MCA *[Le2008]*.

This tools uses MLG samples.

1.4.5 Neighbor-Joining Analysis

This tool generates neighbor-joining trees, constructed from the same genetic distance matrix used in the PCoA analysis. VivaxGEN employs the APE library from the R statistical suite to generate and plot the neighbour-joining tree *[Paradis2004]*.

This tools uses MLG samples.

FILE FORMATS

This guide provides detailed information on file formats for input files.

2.1 Sample Information File Format

The sample information input file contains sample metadata. The format is a tab-delimited text file or comma-separated value text file. The system will deduce the format by the extension of the filename: use .txt, .tab or .tsv for the tab-delimited format, and use .csv for the comma-separated value format.

The list of fields is presented below.

Only the **SAMPLE** field is mandatory, but it is highly recommended to add details on **COUNTRY** and **COLLECTION_DATE**. Please note that some of the fields can only contain *controlled vocabularies*. These are predefined terms set by the systems administrator. The controlled vocabularies are used to maintain consistency between datasets. Current available vocabularies/keywords are listed at the end of this document. If you require a new keyword, please contact the systems administrator at anto@eijkman.go.id.

SAMPLE (mandatory) A unique string (unique within the batch) indicating sample identifier/name. Allowed characters are alphanumerics, dash, dot and underscore. **Do not use space**.

COUNTRY The country where the sample was collected or where the patient presented with the infection. Use the 2-character country code (refer to ISO-3166) or internet identification for consistent data.

ADMINL1 Administrative Level 1 region where the patient presented with the infection (refer to ISO-3166).

ADMINL2 Administrative Level 2 region where the patient presented with the infection.

ADMINL3 Administrative Level 3 region where the patient presented with the infection.

ADMINL4 Administrative Level 4 region where the patient presented with the infection.

COLLECTION_DATE Date at which the patient donated the sample. Must be in format of **YYYY/MM/DD** to be read correctly into the platform.

PASSIVE_DETECTION Y (yes) or N (no).

AGE Patient age in years. Must be an integer.

GENDER F (female), M (male).

BLOOD_WITHDRAWAL (Controlled Vocabularies) String indicating the blood withdrawal method e.g. capillary, venous or other.

BLOOD_STORAGE (Controlled Vocabularies) String indicating the blood storage method e.g. blood tube, dried blood spot or other.

MICROSCOPY_IDENTITY (Controlled Vocabularies) Plasmodium Spp. determined by microscopy. For single species infections: Pf, Pk, Pm, Po, Pv or X (for unknown). For mixed species infections, list the species present in alphabetic order, separated by "/" e.g. Pf/Pv or Pf/Pk/Pv

PCR_IDENTITY (Controlled Vocabularies) Plasmodium Spp. determined by microscopy. Details as for field MICROSCOPY_IDENTITY

PCR_METHOD String indicating the PCR method used for species confirmation.

SYMPTOMATIC_STATUS Indication of whether the patient was symptomatic or not; Y (yes) or N (no).

PARASITE_DENSITY Estimated number of parasites per microliter of blood. Must be an integer.

TYPE [P/R/D] Type of the sample, either P (for population sample), R (for reference sample) or D (for dummy sample). If left blank, the value will be the default, which is P (population). A sample will be included in the population analysis if the type is P, otherwise it will be discarded from the analysis set (unless the user specifically asks to include others)

DAY Sampling day after the first blood collection date. Must be an integer. The default value is 0 (as in day-0). Most population genetic analysis will only use the day-0 samples.

RECURRENT Indication of whether the sample is a recurrence or not; Y (yes) or N (no).

RELATED_SAMPLE If multiple samples have been obtained from the same patient, the sample identifer(s) field SAMPLE of the related sample(s) must be provided.

SUBJECT_CODE Subject code (only fill this if the subject has been saved to the data base previously). This will only be used if there are different samples on different batches that are donated by a same patient (subject).

INT1 Free field that the user may use to add additional sample metadata of interest that is not provided in the above fields. Custom integer.

INT2 Free field that the user may use to add additional sample metadata of interest that is not provided in the above fields. Custom integer.

STRING1 Free field that the user may use to add additional sample metadata of interest that is not provided in the above fields. Custom string.

STRING2 Free field that the user may use to add additional sample metadata of interest that is not provided in the above fields. Custom string.

REMARK Field for providing any general remarks/comments on a given sample.

2.2 FSA Information File Format

The FSA information input file contains information on each FSA file to be uploaded to the database to enable the system to match each FSA file with the correct sample (SAMPLE) and to identify the assays (PANEL) present in each FSA file. The format is a tab-delimited text file or comma-separated value text file. The system will deduce the format by the extension of the filename: use .txt, .tab or .tsv for the tab-delimited format, and use .csv for the comma-separated value format.

The list of fields is presented below.

All fields are mandatory, however **OPTIONS** can be left blank.

SAMPLE A string indicating the sample identifier/name. This field is used to match the FSA files with the corresponding Sample Information and the sample identifiers must therefore be written exactly the same as in the Sample Information file. The system will not upload any FSA files that cannot be matched with an existing SAMPLE string in the Sample Information file.

FILENAME A string indicating the FSA file name. This field is used to match the FSA files with the corresponding FSA Information and the FSA filenames must therefore be written exactly the same as in the FSA zip file.

PANEL A string indicating the panel of assays in a given FSA file. For example, MZ2 is a panel of assays for markers MS5, MS12 and MS20 using LIZ-600 size standard. The panel must be selected from an existing list available in the database: to view this list, select **Browse** from the

drop-down menu and then **Panel**. If your panels or markers are not available on the existing list, please contact the systems administrator at anto@eijkman.go.id.

OPTIONS A string indicating the markers to exclude from a given FSA file, for example if an assay was repeated and data on that assay is therefore present in two or more FSA files to be uploaded to the database. Must be in the format exclude=markername e.g. exclude=MS10. Please use marker names as detailed in the system: to view the list of marker names, select Browse from the drop-down menu and then Markers. If no markers are to be excluded from a given FSA file, leave this field blank.

2.3 Controlled Vocabularies / Keywords

All available, updated controlled vocabularies / keywords can be inspected within VivaxGEN.

[Anderson2000] Anderson TJ, et. al. 2000. Microsatellite markers reveals a spectrum of population structures in the malaria parasite Plasmodium falciparum. *Mol Biol Evol* - PUBMED:11018154

[Excoffier2010] Excoffier L, Lischer HEL. 2010. Arlequin suite ver 3.5: A new series of programs to perform population genetics analysis under Linux and Windows. *Mol Ecol Resour* - PUBMED:21565059

[Haubold2000] Haubold B, Hudson RR. 2000. LIAN 3.0: detecting linkage disequilibrium in multilocus data. *Bioinformatics* - PUBMED:11108709

[Hedrick2005] Hedrick PW. 2005. A standarized genetic differentiation measure. *Evolution* - PUBMED:16329237

[Jost2008] Jost L. 2008. G(ST) and its relatives do note measure differentiation. *Mol Ecol* - PUBMED:19238703

[Le2008] Le S, Josse J, Husson F. 2008. FactoMineR: An R package for multivariate analysis. *Journal of Statistical Software* - DOI:10.18637/jss.v025.i01

[Paradis2004] Paradis E, Claude J, Strimmer K. 2004. APE: Analyses of phylogenetics and evolution in R langueage. *Bioinformatics* - PUBMED:14734327
